# In Prostate Cancer Cells Cytokines Are Early Responders to Gravitational Changes Occurring in Parabolic Flights

**DOI:** 10.3390/ijms23147876

**Published:** 2022-07-17

**Authors:** Herbert Schulz, Dorothea Dietrichs, Markus Wehland, Thomas J. Corydon, Ruth Hemmersbach, Christian Liemersdorf, Daniela Melnik, Norbert Hübner, Kathrin Saar, Manfred Infanger, Daniela Grimm

**Affiliations:** 1 Department of Microgravity and Translational Regenerative Medicine, Medical Faculty, University Hospital Magdeburg, Otto von Guericke University, Universitätsplatz 2, 39106 Magdeburg, Germany; herbert.schulz@med.ovgu.de (H.S.); dorothea.dietrichs@st.ovgu.de (D.D.); markus.wehland@med.ovgu.de (M.W.); daniela.melnik@med.ovgu.de (D.M.); manfred.infanger@med.ovgu.de (M.I.); 2 Clinic for Plastic, Aesthetic and Hand Surgery, Medical Faculty, University Hospital Magdeburg, Otto von Guericke University, Leipziger Straße 44, 39120 Magdeburg, Germany; 3 Research Group ‘Magdeburger Arbeitsgemeinschaft für Forschung unter Raumfahrt- und Schwerelosigkeitsbedingungen’ (MARS), Otto von Guericke University, Universitätsplatz 2, 39106 Magdeburg, Germany; 4 Department of Biomedicine, The Faculty of Health, Aarhus University, Ole Worms Allé 4, 8000 Aarhus, Denmark; corydon@biomed.au.dk; 5 Department of Ophthalmology, Aarhus University Hospital, Palle Juul-Jensens Blvd. 99, 8200 Aarhus, Denmark; 6 Gravitational Biology, Institute of Aerospace Medicine, German Aerospace Center, Linder Höhe, 51147 Cologne, Germany; ruth.hemmersbach@dlr.de (R.H.); christian.liemersdorf@dlr.de (C.L.); 7 Cardiovascular and Metabolic Sciences, Max Delbrück Center for Molecular Medicine in the Helmholtz Association (MDC), 13125 Berlin, Germany; nhuebner@mdc-berlin.de (N.H.); ksaar@mdc-berlin.de (K.S.); 8 DZHK (German Centre for Cardiovascular Research), Partner Site Berlin, 10785 Berlin, Germany; 9 Charité-Universitätsmedizin, 10117 Berlin, Germany

**Keywords:** parabolic flight, microgravity, hypergravity, prostate cancer, PC-3, RNA sequencing, qPCR, cytokines, non-coding

## Abstract

The high mortality in men with metastatic prostate cancer (PC) establishes the need for diagnostic optimization by new biomarkers. Mindful of the effect of real microgravity on metabolic pathways of carcinogenesis, we attended a parabolic flight (PF) mission to perform an experiment with the PC cell line PC-3, and submitted the resulting RNA to next generation sequencing (NGS) and quantitative real-time PCR (qPCR). After the first parabola, alterations of the F-actin cytoskeleton-like stress fibers and pseudopodia are visible. Moreover, numerous significant transcriptional changes are evident. We were able to identify a network of relevant PC cytokines and chemokines showing differential expression due to gravitational changes, particularly during the early flight phases. Together with differentially expressed regulatory lncRNAs and micro RNAs, we present a portfolio of 298 potential biomarkers. Via qPCR we identified *IL6* and *PIK3CB* to be sensitive to vibration effects and hypergravity, respectively. Per NGS we detected five upregulated cytokines (*CCL2*, *CXCL1*, *IL6*, *CXCL2*, *CCL20*), one zink finger protein (*TNFAIP3*) and one glycoprotein (*ICAM1*) related to c-REL signaling and thus relevant for carcinogenesis as well as inflammatory aspects. We found regulated *miR-221* and the co-localized lncRNA *MIR222HG* induced by PF maneuvers. *miR-221* is related to the PC-3 growth rate and *MIR222HG* is a known risk factor for glioma susceptibility. These findings in real microgravity may further improve our understanding of PC and contribute to the development of new diagnostic tools.

## 1. Introduction

Prostate cancer (PC) is the second leading cause of cancer death in the US male population after lung cancer. The American Cancer Society predicts 268,490 new cases of PC in the US in 2022, which is about 0.165% of the male population. The disease, which primarily occurs at an older age, has a predictable treatment prognosis. However, the 5-year survival prognosis is drastically reduced from 100% to 31% when metastases ensue (The American Cancer Society). Accordingly, early management of PC is a challenging matter in medicine. In the context of personalized medicine, transcriptional studies of factors influencing prostate carcinogenesis and progression may add new early-onset biomarkers to the diagnostic portfolio.

In general, cancer cells are characterized by uncontrolled proliferation, elevated survival and activated invasion potential. The genetic and epigenetic alterations lead to an impairment of the cell death mechanisms that are initiated upon sustained damage to the cell. As we know from previous studies, gravitational changes have a lasting effect on tumorigenesis [1,2] including cytoskeletal and focal adhesion changes and alterations in differentiation and proliferation [3,4,5,6,7,8], increased apoptosis [5,9,10], the release of cytokines [11], altered interleukin, OPN, TLN1, and CTGF signaling [9], and influenced PI3K/AKT/mTOR (PAM) signaling [7,8], to only name the most significant ones. Furthermore, a previous study by Corydon and coworkers also showed that gravitational changes have specific effects on the F-actin-cytoskeleton in non-carcinogenic epithelium cell lines [12].

In previous studies, DU-145 and PC-3 prostate carcinoma cell lines were exposed to simulated microgravity (s-µ*g*) for several days using a high aspect rotating-wall vessel (HARV) or random positioning machine (RPM), respectively [13,14,15,16]. DU-145 prostate cancer cultures revealed a slowed growth, an increase in cytokeratin 8 and 18, together with an increase in cell–cell and cell–matrix interactions, and ultimately an organotypic 3D spheroid organization after a few hours under HARF influence compared to the static 1*g* control [13,14]. Furthermore, significant changes in VEGF signaling were detected in aggressive PC-3 prostate carcinoma cells, resulting in early (4 h s-µ*g*) and late (3 days to 5 days s-µ*g*) downregulation of *VEGFA* expression [15,16], indicating attenuation of PC-3 aggressiveness [15]. In contrast, a significant increase in *IL6* and *CXCL8* gene expression in the first 24 h of s-µ*g* hints towards a more aggressive phenotype of PC-3 [16]. Moreover, substantial components of PAM signaling were deregulated after a 5-day RPM exposure and *AKT1* was upregulated in multi-cellular spheroids, but even more in adherent cells [15]. Consequently, AKT kinase inhibitors are already being assessed in phase 3 clinical trials and seem to be particularly effective in castration-resistant metastatic prostate cancer [17,18], which supports the idea that µ*g*-based research is relevant for increasing the current knowledge about cancer progression and metastases on Earth.

In summary, it was found in previous studies that crucial key positions of carcinogenesis are influenced by microgravity and various *g* sensitive key players tend to promote a milder progression. Since the s-µ*g* influence on already known major actors of prostate carcinogenesis have been investigated, the idea of our current study was to identify transcriptome-wide early responders to gravitational changes in PC-3 using a parabolic flight scenario. Parabolic flights are a cost-effective and proven approach to briefly expose cell lines to gravitational changes. During a parabolic flight (PF), which typically consists of 31 parabolas, samples experience repeating phases of free fall and hypergravity [19]. In a PF, each parabola consists of short periods (22 s) of ~0.01*g* (microgravity), encased by 1.8*g* (hypergravity) phases. These alterations allow tracking of dynamic transcriptional responses of known biomarkers of prostate cancer development and identification of, for the first time in prostate carcinoma cells, novel µ*g* related biomarkers by transcription-wide RNA sequencing. To distinguish microgravity from other influences occurring during parabolic flight, the expression of key genes of the extracellular matrix, focal adhesion, PAM signal transduction, cell growth and apoptosis were additionally studied under hypergravity conditions (1.8*g*) and vibrations as they occur during a typical parabolic flight. Focusing on early responders reduces the accumulation of secondary expression changes. Finally, both transcriptional primary effects and secondary effects are relevant to research. In a biomedical context, early responses to the gravitational stimulus are closer to the cause, i.e., genetic epigenetic constitution, and subsequent secondary effects are closer to physiological, morphological changes, e.g., progression of cancer. In particular, the study of the first parabola opens the possibility to observe the primary transcriptional response to gravitational changes before the point of divergence of related signaling pathways, mostly unaffected by later secondary regulatory responses, to unravel the origin of gravitational metabolic networks. In conclusion, the knowledge of the primary transcriptional responses to gravitational changes is essential for assessing subsequent synergistic and competitive effects of prostate carcinogenesis under µ*g*.

## 2. Results

### 2.1. Gravitational Changes of the F-Actin Cytoskeleton

When comparing the F-actin staining under 1*g*, first parabola (1P) and 31st parabola (31P) conditions, it is noticeable that stress fibers appear already after the first parabola, but formation of pseudopodia can be observed only sporadically (Figure S1A,B). Nevertheless, the observed effects after 1P are more pronounced than in our previous PF experiment with human chondrocytes [20]. After 31 parabolas the stress fibers are more frequent and more pronounced; the most striking change though is an increase of pseudopoidia and lamellipodia (Figure S1C).

### 2.2. qPCR Results

**Figure 1:**
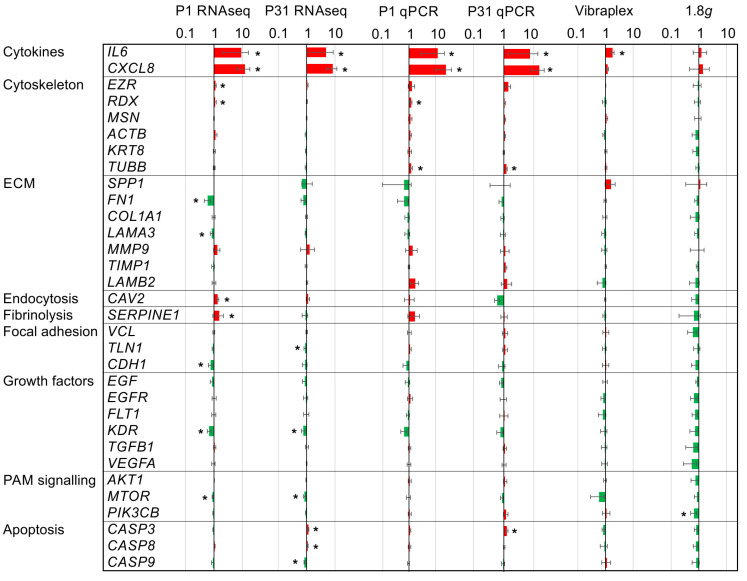
Gene expression ratios of 32 key players of carcinogenesis in PC-3 prostate cancer cells under altered gravitational conditions measured by qPCR and RNAseq: Results determined after P1, after P31, after vibration-exposure and after hypergravity (1.8*g*)-exposure in relation to the respective 1*g* control samples. The red color indicates upregulated genes and the green color downregulated genes. Significant changes (nominal *p* < 0.05) are indicated by asterisks. The ratios are given on a logarithmic scale.

To isolate µ*g*-related expression changes, the main intention of the qPCR analysis was to compare RNA expression changes in PF with those under hypergravity (1.8*g*) and vibration. Additionally, corresponding results of the RNAseq experiment (control vs. 1P and control vs. 31P) were added to check the validity of the qPCR results. Non-concordant results between qPCR and RNAseq are significantly enriched in genes with weak regulation (<2-fold) [21]. In contrast to Section 2.3, the candidate gene approach in this section also considers the differential expression with less than two-fold difference. Accordingly, a cross-validation of qPCR results with RNAseq results seemed appropriate. First, it should be noted that under hypergravity (*PIK3CB*) and vibration (*IL6*) exposure, only 1 of the 32 investigated genes was differentially expressed (Figure 1).

#### 2.2.1. Cytokines

The qPCR study served to assess the influence of PF and its sub-aspects, vibration and hypergravity, on key players of carcinogenesis. Strikingly, the cytokines *IL6* and *CXCL8* were markedly upregulated as measured after the 1P and 31P (RNAseq and qPCR, Figure 1). However, the more moderate but significant upregulation of *IL6* in the vibration experiment indicates that IL6 regulation is at least in part due to aircraft vibration.

#### 2.2.2. Cytoskeleton 

Of the six cytoskeletal genes tested via qPCR, only radixin (*RDX*) and tubulin, beta class I (*TUBB*), are differentially expressed between the 1*g* control and 1P. *TUBB* is additionally differentially expressed between control and 31P. However, *TUBB* qPCR results cannot be confirmed by RNAseq results, but *TUBB* is in RNAseq nominally significant between 1P and 31P (*p* = 0.009). TUBB is a structural component of microtubules and forms a dimer with alpha tubulin in this function. In RNAseq, two components of alpha tubulin *TUBA4A* and *TUBA1B* are significant differentially expressed after multiple testing corrections in comparison between 1P and 31P (P_adj_ = 0.004 and 0.023 respectively). RDX probably possesses a role in coupling actin to the plasma membrane. Actin itself is not differentially expressed in the RNAseq experiment. 

#### 2.2.3. PI3K/AKT/mTOR (PAM) Signaling

The phosphoinositide-3-kinase, catalytic, beta polypeptide (*PIK3CB*) is a catalytic subunit of phosphoinositide 3-kinase (*PI3K*). *PIK3CB* is not regulated in PF, but four genes encoding catalytic subunits of PI3K are deregulated between 1*g* and 1P (PIK3CA, P_adj_ = 2.8 × 10^−5^; *PIK3R3*, P_adj_ = 2.8 × 10^−5^; *PIK3R1*, P_adj_ = 1.9 × 10^−4^; *PIK3C2A* P_adj_ = 2.8 × 10^−3^). It should be noted that the phosphoinositide 3-kinase expression is modulated by hypergravity (at least in one subunit) whereas the gene expression of other subunits is susceptible to gravitational changes in PF.

#### 2.2.4. Apoptosis

The qPCR assessment measuring significant *CASP3* upregulation after 31P is confirmed by RNAseq and complemented by a significant upregulation of *CASP8* and significant downregulation of *CASP9* in the RNAseq analysis. Regulations of the CASP genes are moderate and do not reach a two-fold upregulation or downregulation, respectively. Additionally, two upstream key genes of the apoptosis cascade, namely the gene coding the Tumor Necrosis Factor Receptor Type 1 (*TRADD*, P_adj_ > 0.7) and the gene coding the Fas Associated Via Death Domain (*FADD*, P_adj_ > 0.1), are not regulated by PF in PC-3 cells.

#### 2.2.5. RNAseq to qPCR Comparison

As expected, additional genes could be detected in the RNAseq experiment as being regulated in PF due to the increased sensitivity (Figure 1). However, more importantly, apart from TUBB, all other qPCR PF findings could be confirmed. Apart from the cytokines *IL6* and *CXCL8*, none of the remaining 30 qPCR-tested genes achieved an adjusted significant two-fold change in the RNAseq control to 1P comparison or in the RNAseq control to 31P comparison. The genes *RDX*, *SERPINE1*, *CDH1* and *MTOR* are only nominally significant in the control to 1P comparison, as is *KDR* in the control to 31P comparison.

### 2.3. RNAseq Analysis

#### 2.3.1. QC

Demultiplexed FASTQ files are used to process the 10 PF and 5 control samples individually in the secondary analysis using STAR. Fortunately, all samples exceeded a mean quality score of 35; the ratio of Q30 bases was, with one exception, >93% and the ratio of perfect barcodes >98% (Table S1). No significant difference between controls and PF samples for any of the parameters was observed.

#### 2.3.2. General Expression Profile Reveals a Potential Habituation Effect

**Figure 2:**
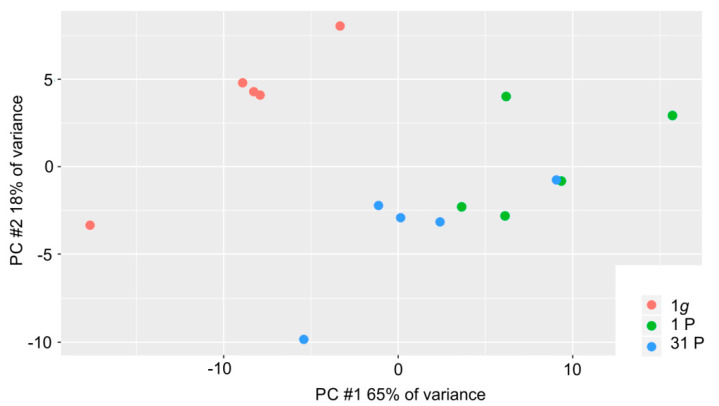
Distribution of the 15 PC-3 samples in the first two principal components; 1*g* control samples, 1P and 31P inflight samples are color-coded in red, green and blue, respectively.

The general expression profile was determined using principal component analysis on the read counts. We found a high proportion of variance in the first two principal components (PCs, 65% of the variance in PC #1 and 18% of the variance in PC #2, Figure 2). This is due to the low sample number (*n* = 15) and to a strong effect of microgravity on the PC-3 cells. This strong effect is understandable if one considers the changes of the F-actin cytoskeleton during the PF as shown in Figure S1. The three conditions are incompletely separated in PC #1 with a tending intermediate position of the measurements after 31P. This suggests a habituation effect of the PC-3 cells to the alternating gravitational conditions during the flight.

#### 2.3.3. Differential Expression Analyses Reveal Expression Difference in Cytokines and Genes of the NFκB System Induced by Gravitational Changes

**Figure 3:**
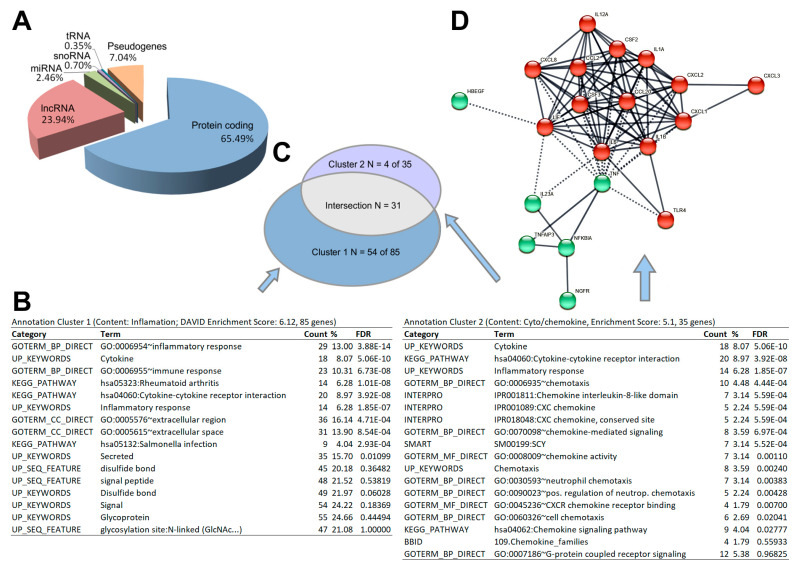
Results of the post hoc analyses on 298 genes. (**A**) Functional assembly of differentially expressed genes. (**B**) Result of the annotation clustering. The two most stringent clusters with an enrichment score > 5 are shown. (**C**) Venn diagram of the two functional enrichment clusters. (**D**) STRING protein–protein interactions of annotation cluster 2 genes.

By comparing the number of genes significantly expressed until the end of 1P with the number of genes significantly expressed after 31P, the data, in terms of both stringency and fold change, reveal that the strongest changes occur in the early flight phase. DESeq2 results for the set of 298 differentially expressed genes and their dotplot visualization can be downloaded (Table S2, Figure S2). The total sum of 298 two-fold differentially expressed genes (DEGs) at P_adj_ less than 0.05 is divided (with overlaps) into 208 DEGs between 1*g* and post 1P, 129 DEGs between 1*g* and post 31P, 47 DEGs between post 1P and post 31P. In addition to the chemokine encoding *CXCL2*, a significant > two-fold upregulation after P1 is followed by a significant > two-fold downregulation after P31 for another eight genes (*ATOH8*, *AC107308.1*, *AC018628.2*, *KRTAP1-5*, *MIR221*, *CCN2*, *ZEB2*, *AC008264.2*). Even though the applied RNAseq method enriches coding genes by a poly-A selection step, we found that the DEG set contains a considerable number of long non-coding RNAs (23.9% lncRNAs) and micro RNAs (2.46% miRNAs) in addition to 65.5% coding genes (Figure 3A).

The functional enrichment analyses over the annotations included in DAVID (GO, KEGG, Interpro, Smart, etc.) revealed two annotation clusters with an enrichment score of >5. The gene clusters comprise 85 genes (cluster 1) and 35 genes (cluster 2) with a strong overlap of 31 genes between the two clusters (Figure 3B,C). The annotations suggest a strong (over-)representation of cytokines (both clusters, FDR = 5.06 × 10^−10^) in those interactions (hsa04060; both clusters, FDR = 3.92 × 10^−8^), inflammatory genes (GO:0006954; cluster 1, FDR 3.88 × 10^−14^), chemokines and chemotaxis (IPR001089 and GO0006953; cluster 2, FDR = 5.59 × 10^−4^, FDR = 4.48 × 10^−4^ respectively). Visualization of the pairwise protein interaction of cluster 2 using the EMBL STRING database with the highest confidence (score > 0.9) reveals a core with C-X-C motif and C-C motif chemokines surrounded by interleukins and other cytokines.

## 3. Discussion

**Figure 4:**
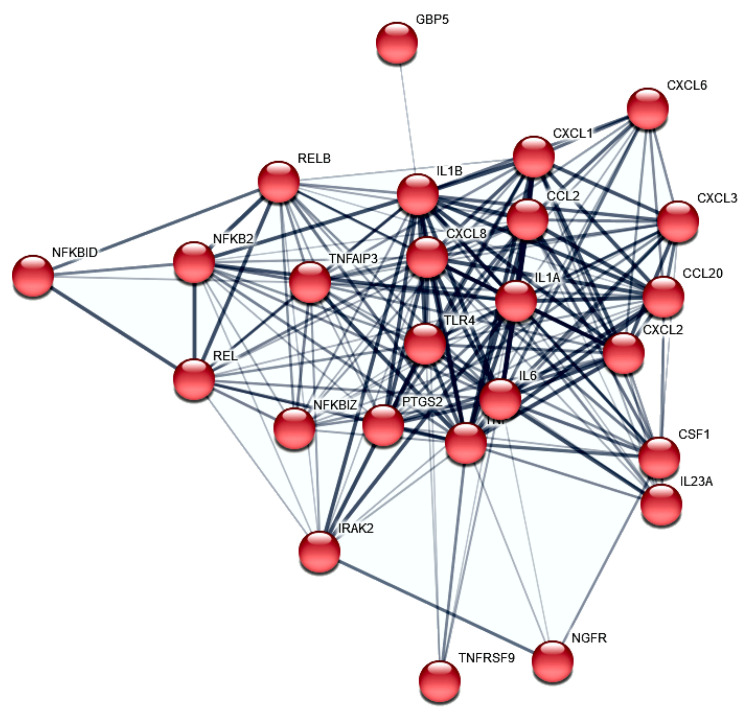
STRING protein–protein interactions of PC-3 PF differential expressed genes. The 23 protein to protein interactors (confidence > 0.4) annotated in cluster 1 and involved in the inflammatory response (GO:0006954) are shown.

A PF is a long-established platform acknowledged by NASA (National Aeronautics and Space Administration), ESA (European Space Agency) and other national space agencies for studying molecular changes under the influence of gravitational changes. This platform to study the early effects of real microgravity has been used for molecular studies on, among others, Euglena gracilis [22], mouse stem cells [23] and human cardiomyocytes [24], chondrocytes [20,25], endothelial cells [26,27], but especially in cancer cells [6,8,28,29]. The parabolic flight campaign opened-up the possibility for us to generate a list of 298 gravisensitive PC-3 genes and categorize them by subsequent enrichment and interaction network analyses. However, the specific carcinogenesis reference is crucial for the usability of the study. We were able to determine an accumulation of cytokines (DAVID FDR 5.06 × 10^−10^, Figure 3) and, in particular, chemokines (DAVID FDR 5.59 × 10^−4^). These are, in turn, partially integrated into the portfolio of genes of the inflammatory response (DAVID FDR = 3.88 × 10^−14^, Figure 4).

### 3.1. NF-kB Signaling and Chemokines

**Figure 5:**
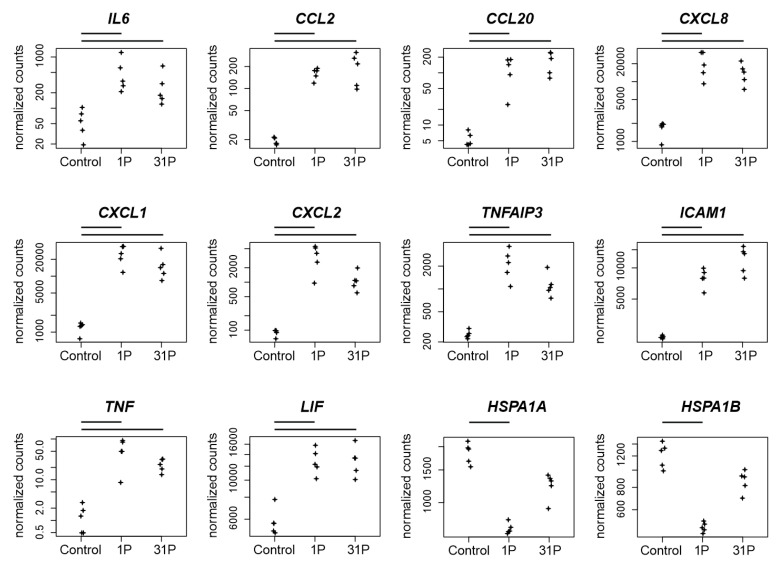
Normalized PF RNAseq counts of twelve candidate genes over the conditions control, 1P and 31P. Normalized mapped RNAseq read counts to the genes encodes the cytokine *IL6*, two C-C motif chemokines (*CCL2*, *CCL20*), three C-X-C motif chemokines (*CXCL8*, *CXCL1*, *CXCL2*), the zinc finger protein *TNFAIP3*, the glycoprotein *ICAM1*, the proinflammatory cytokine *TNF*, the pleiotropic cytokine *LIF* and the heat shock 70 kDa protein (*HSPA1A*, *HSPA1B*). Bars on top of the graph indicate two-fold significant (P_adj_ < 0.05) differential expression between the conditions.

Because the post parabola showed one significant upregulation of the *RELB* proto-oncogene NF-KB subunit (P_adj_ to 1*g* = 1.3 × 10^−37^), which is accompanied by the regulation of NF-KB-related inflammatory genes, a detailed consideration of these relationships is appropriate. de Jesús and Ramakrishnan have shown that a c-Rel knockout in mouse embryonic fibroblasts (MEF) leads to an upregulation of *CCL2*, *CCL7*, *IP-10* (*CXCL10*), *CXCL1*, *A20* (*TNFAIP3*), *IL-6*, *CXCL2*, *CCL20*, and *ZFP36* by maintaining the expression kinetics [30]. Additionally, they found *ICAM1* and *VCAM1* upregulated with slowed kinetics. The gene *REL* encodes c-REL and was significantly upregulated after parabola one (P_adj_ to 1*g* = 7.7 × 10^−16^) in our PF RNAseq experiment. Notably, seven of the eleven genes mentioned by de Jesús and Ramakrishnan as c-REL knock-out-dependently upregulated [30] are also highly significant and more than two-fold upregulated after P1 in our PC-3 PF (Figure 5): *CCL2* (P_adj_ to 1*g* = 5.0 × 10^−49^), *CXCL1* (P_adj_ to 1*g* = 2.8 × 10^−50^), *TNFAIP3* (P_adj_ to 1*g* = 9.5 × 10^−23^), *IL6* (P_adj_ to 1*g* = 4.0 × 10^−6^), *CXCL2* (P_adj_ to 1*g* = 5.2 × 10^−33^), *CCL20* (P_adj_ to 1*g* = 3.1 × 10^−17^), and *ICAM1* (P_adj_ to 1*g* = 1.7 × 10^−52^), whereas the joint significant upregulation of *CCL2* and *RELB* after the first parabola is in concordance with the *CCL2* expression depletion described by de Jesús and Ramakrishnan in the RELB MEF knock out experiment [30].

In conclusion, although c-Rel and RELB are important factors in the regulatory cascade, the presence of other regulatory key players must be assumed. Furthermore, we were able to demonstrate the activation of genes relevant to inflammation by the gravitational change in PC-3.

### 3.2. Cytokines and Chemokines

The inflammatory cytokines TNF-α and LIF influence the CXCL-8 expression in pancreatic cancer progression [31]. CXCL-8 has been detected in prostate epithelium and stroma, is involved in multiple intracellular signaling [32] and upregulates the activity of Akt kinase [33]. *TNF*, *LIF* and *CXCL8* are upregulated after parabola one in our PC-3 PF (P_adj_ to 1*g* = 8.6 × 10^−12^, 1.9 × 10^−15^ and 2.0 × 10^−22^, respectively). In PC-3 cells, migration and proliferation are stimulated by *CXCL3* overexpression [34]. In general, a highly increased secretion of the chemokine CXCL3 can be detected in PC-3 while an elevated expression of *CXCR2* was observed in the PC cell lines DU145, LNCaP and exogenous CXCL3, which does not affect proliferation but migration [35]. The experimental autoimmune prostatitis (EAP) mice are a model for the autoimmune dysfunction of chronic prostatitis. In EAP prostate, the proinflammatory cytokine interleukin 17 and the chemokine ligands *CXCL1* and *CXCL2* are upregulated. These findings suggest that IL-17 promotes the production of *CXCL1* and *CXCL2* [36]. In support of this notion, we find *CXCL1* and *CXCL2* to be upregulated after P1 (P_adj_ to 1*g* = 2.8 × 10^−50^ and 5.2 × 10^−33^, respectively).

### 3.3. Protein Transport

The two 70 kDa heat shock protein coding genes *HSPA1A* and *HSPA1B* are both downregulated after P1 (P_adj_ to 1*g* = 4.6 × 10^−43^ and 2.5 × 10^−33^ respectively, Figure 5). The proteins have chaperone and lipid-binding functions and are involved in protein stabilization and folding. The gene *HspA1B* is one of the most prominent biomarkers for PC [37] and *HSPA1A* and *HSPA1B* upregulation is associated with a poor survival in colon cancer [38]. Furthermore, HspA1A specifically inhibits the malignant progression of Arid2-deficient lung cancer [39]. The known translocation of HspA1A toward the plasma membrane and the resulting presentation at the cell surface [40] are possible targets of gravitational variation in our PF experiment.

### 3.4. Non-Coding RNA

Although the applied RNAseq method is targeted towards coding genes by poly-A filtering, 23.9% and 25% of significantly and two-fold regulated genes are lncRNAs and miRNAs, respectively (Figure 3A). miRNAs and lncRNAs are important regulators of translation and transcription, respectively, so it is interesting to investigate whether expression of the non-coding genes were altered early on by microgravity.

**Figure 6:**
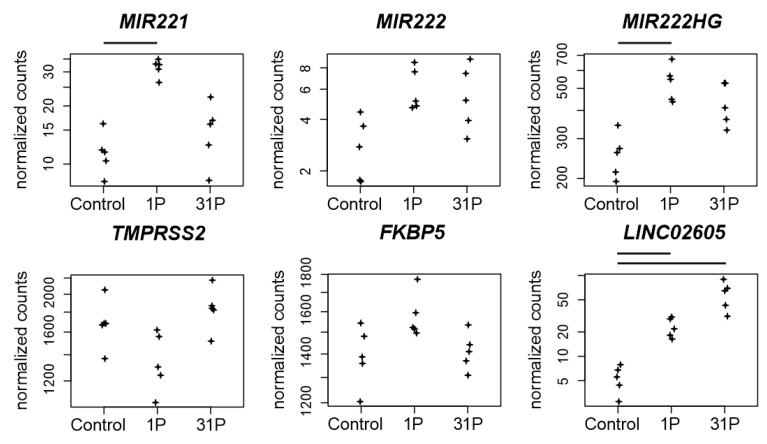
Normalized PF RNAseq counts of four non-coding RNAs and two protein coding RNAs. Expressions of the micro RNAs *miR-221* and *miR-222*, of the lncRNA *MIR222HG*, of the serine protease encoding *TMPRSS2* gene, of the Peptidyl-Prolyl Cis-Trans Isomerase encoding gene FKBP5 and of the lncRNA *LINC02605* are given. Bars on top of the graph indicate two-fold significant (P_adj_ < 0.05) differential expression between the conditions.

The role of *miR-221/222* in PC remains controversial but upregulation of *miR-221/222* in CRPC is associated with an androgene receptor modulation during cancer development [41]. It has been shown that elimination of *miR-221* by CRISPR in PC-3 cells led to a reduced growth rate and expression of cell-cycle genes [42] through post transcriptional inhibition of *CDKN1B*/p27 [43]. In cells fixed after P1, we found a significantly increased miR-221 expression (q = 1.65 × 10^−5^) and at the same time expression depletion of *CDKN1B* (P_adj_ to 1*g* = 5.07 × 10^−5^). Upstream of *miR221/222 MIR222HG*, a long non-coding RNA (lncRNA), is located. *MIR222HG* is associated with PC progression to CRPC, increases androgen-independent cell growth and represses the expression of *TMPRSS2* and *FKBP5* in hormone-sensitive prostate cancer [44]. We found a significant increase in *MIR222HG* expression in cells fixed after first parabola (P_adj_ to 1*g* = 1.47 × 10^−9^), but no corresponding expression depletion in *TMPRSS2* and *FKBP5* (Figure 6).

*MIR3142HG*, a long non-coding RNA (lncRNA) associated with genomic instability, is upregulated in PC-3 cells in PF after the first parabola (P_adj_ to 1*g* = 3.7 × 10^−34^, Figure 6). Its role in cancer progression is unclear. *MIR3142HG* is propagated as a risk factor for glioma susceptibility in the Chinese Han population [45], but it is also assumed to serve as a protective factor for head and neck squamous cell carcinoma (HNSCC) because the upregulated expression of *MIR3142HG* was associated with better survival outcomes [46].

The lncRNA *LINC02605* is involved in the antiviral immune response and its knockdown leads to an increased viral replication [47]. *LINC02605* is upregulated in PC-3 cells after the first parabola (P_adj_ to 1*g* = 2.9 × 10^−6^) and even more after parabola 31 (P_adj_ to 1*g* = 1.9 × 10^−16^). It has an effect on *hsa-miR-107* regulation on the protein expression of phosphatase and tensin homolog (PTEN) [47,48]. Both miR-107 and *PTEN* are important players in carcinogenesis. miR-107 has a potential role in regulating NEDD9 in the invasion, migration and proliferation of breast cancer [49]. Additionally, miR-107 is hypothesized to deactivate the phosphatidylinositol 3-kinase (PI3K)/Akt pathway in a hypopharyngeal squamous cell carcinoma (HSCC) study [48]. Downregulation of *miR-107* by the potential therapeutic target LINC-DUBR suppressed malignant progression of ovarian cancer [50].

### 3.5. qPCR: Apoptosis, Hypogravity, and Vibration

**Figure 7:**
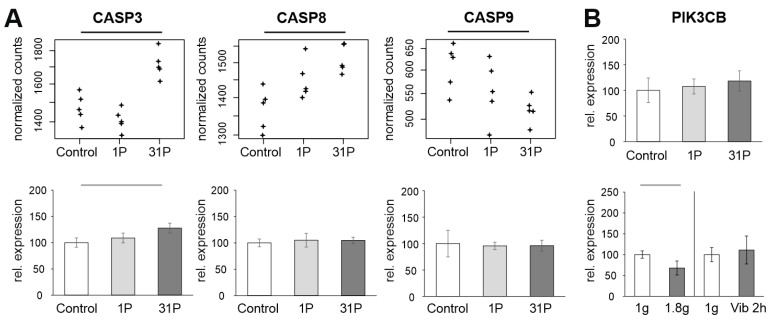
Analysis of apoptosis-related genes in PF samples. (**A**): Normalized PF RNAseq counts and qPCR ratios of the three apoptosis-related genes *CASP3*, *CASP8* and *CASP9*. (**B**): *PIK3CB* qPCR expression relative to the controls of the PF, hypergravity and Vibraplex experiment. Black and grey bars on top of the graph indicate adjusted and nominal significant (*p* < 0.05) differential expression, respectively.

The set of genes to be validated by qPCR shows that a substantial fraction of PC key players is unaffected by the gravitational influence of PF. However, it should be noted that the cytokine interleukin 6 is at least partially upregulated due to the vibration effect. Since cytokines play a major role in early PF-induced regulation, *IL6* requires further investigation. Furthermore, apoptotic processes seem to start at 31P. Although the effects on *CASP3, CASP8*, and *CASP9* are at the limit of detection even with RNAseq, mutual validation of RNAseq and qPCR for upregulation of *CASP3* indicates an albeit weak but stable finding (Figure 7A). Since more than 31P are not performed for technical reasons, it should be sufficient to keep an eye on the apoptosis problem. The downregulation of *PIK3CB* after 24 h of simulated microgravity obtained by random positioning published in our recent paper [16] does not technically match the downregulation measured in the present study under hypergravity (Figure 7B). *PIK3CB* following regulation cascades should therefore be kept in mind at least for long-term experiments with preceding hypergravity (e.g., rocket launch during ISS missions). It remains to be verified whether *PIK3CB* is subject to a general sensitivity to gravitational changes.

## 4. Materials and Methods

### 4.1. Cell Culturing

The PC-3 cell line (ECACC 90112714) was purchased from the European Collection of Authenticated Cell Cultures (ECACC). The cells originated from a 62-year-old male Caucasian who suffered from a grade 4 prostate adenocarcinoma.

During cell cultivation, Ham’s F-12 Nutrient Mixture (F-12) medium (Gibco, Fisher Scientific, Schwerte, Germany) was used and was supplemented with 10% FCS (Sigma Aldrich, Steinheim, Germany) and 1% penicillin/streptomycin (Life Technologies, New York, USA). The cells were cultured in T75 cm² flasks (Sarstedt, Nümbrecht, Germany). Every 3–4 days the medium was changed. The cells were split using 0.05% trypsin-EDTA (Gibco, Life Technologies, Paisley, UK).

### 4.2. Parabolic Flight Campaign

The 34th PF campaign was organized by the French company Novespace at the Bordeaux-Mérignac airport on behalf of the DLR (Deutsches Zentrum für Luft- und Raumfahrt) as part of their scientific and technological research programs. 

The PF campaign consisted of a total of three flight days with the same experimental setup. The PF was carried out with the Airbus A310 ZERO G (Figure S3A,B) and took about two hours. When the flight altitude was reached, a total of 31 parabolas were flown. A parabola consists of a 20-s climb phase during which the acceleration due to gravity is almost twice as high (1.8*g*). This is followed by the actual 22-s-long µ*g*-phase. In the nosedive, a 20-s phase of hyper-*g* (1,8*g*) follows again. During the PF, the PC-3 cells were fixed after the first and after the 31st parabola using RNAlater (Invitrogen by Thermo Fischer Scientific, Vilnius, Lithuania), thus stabilizing the RNA until isolation.

T75 cm² flasks (Sarstedt, Nümbrecht, Germany) were placed on board in an incubator preheated to 37 °C 1 h before take-off. This was secured inside the so-called flight rack (Figure S3C). The flasks were connected to syringes via a flexible tube and a three-way valve (Figure S3D). The syringes contained 50 mL RNAlater. At the time points mentioned above, the syringes were manually actuated so that the culture flasks were filled with RNAlater at a ratio of approximately 4:1 (RNAlater:medium). Post-flight, the cells were transferred into 15 mL Falcon™ tubes (Sarstedt, Nümbrecht, Germany) by scratching the cells with 25 cm cell scrapers (Sarstedt, Nümbrecht, Germany) and stored suspended in RNAlater at 4 °C until RNA isolation.

For F-actin staining, 200,000 cells were pipetted into eight slideflasks (Thermo Scientific, Waltham, MA, USA) and were incubated for 1.5 days. Of these, four slideflasks remained under 1*g* conditions and served as ground controls. The other four flasks were on board the aircraft in the incubator. Cells were fixed at the designated times using 4% paraformaldehyde (PFA, Sigma-Aldrich, St. Louis, MO, USA) in phosphate buffered saline (PBS; Gibco, Life Technologies, Paisley, UK). The 1*g* ground controls were also fixed at approximately the same times using 4% PFA. The slideflasks remained in 4% PFA until staining.

### 4.3. RNA Isolation

The cell suspension in the 15 mL Falcon™ tubes was diluted 1:5 with PBS. After centrifuging the dilution, the supernatant was discarded. Then, the RNA was isolated using the RNeasy Mini Kit (Qiagen, Hilden, Germany). Then, RTL lysis buffer was added, and the cell pellet was dissolved by pipetting up and down with a syringe. Afterwards, 350 µL of a 70% ethanol dilution was added, 700 µL of the solution was transferred to RNeasy Mini spin column, and the column was centrifuged for 15 s at 8000× *g*. Next, 700 µL RW1 buffer and 500 µL RPE buffer were added and centrifuged in between. Then, 35 µL RNAse-free water was pipetted directly to the spin column membrane and the column was put into a new Eppendorf tube. Finally, the spin column was centrifuged again to detach the RNA from the membrane. 

The quality and the concentration of the RNA of each sample was evaluated by the Nanodrop technique and a spectrophotometer.

### 4.4. Quantitative Real-Time Polymerase Chain Reaction

A 7500 Fast Real-Time PCR System (Applied Biosystems, Darmstadt, Germany) was used both for cDNA synthesis and the following quantitative real time polymerase chain reaction (qPCR). 

First, the RNA was converted into cDNA with the High-Capacity cDNA reverse Transcription Kit (Applied Biosystems, Darmstadt, Germany) on a 96-well plate. Then, 2 µL RT buffer, 0.8 µL dNTP mix, 2 µL RT random primer, 1 µL reverse transcriptase and 4.2 µL nuclease free water were added to each well. In the end, the RNA of each sample was added to get a total amount of 1 µg RNA in 20 µL solution. 

The qPCR was performed with FAST SYBR™ Green Master Mix (Applied Biosystems, Foster City, CA, USA) on a 96-well plate. Every sample was measured in triplicate. Each well contained 1 µL cDNA of the corresponding sample, 7.5 µL SYBR Green Master Mix, 0.042 µL primer forward, 0.042 µL primer reverse and 6.41 µL water (Table S3). The plate was placed in the qPCR instrument and went through the following thermal cycles: 95 °C for 20 s, 95 °C for 3 s, and 60 °C for 30 s. 

The samples were analyzed with the comparative threshold cycle (ΔΔC_T_) method, using the 18 s housekeeping gene as a reference.

### 4.5. F-Actin Staining

F-actin was visualized by means of rhodamine-phalloidin staining (Molecular Probes^®^, Eugene, OR, USA). Moreover, the nuclei were stained with 4′,6-diamidino-2-phenylindole (DAPI, Sigma-Aldrich, Merck Life Science A/S, Søborg, Denmark). The protocol was published earlier in [20].

### 4.6. Microscopy

The slideflasks were investigated using confocal laser scanning microscopy. The observations were made with a Leica DM 2000 microscope and an objective with a 400× magnification. The microscope was also connected to an external light source Leica EL 6000 (Leica Microsystems GmbH, Wetzlar, Germany).

### 4.7. Hypergravity

The hyper-*g* experiments were performed using the DLR Multi-Sample Incubator Centrifuge (MuSIC, DLR, Department of Gravitational Biology) as already described by Aleshcheva et al. [20]. For this purpose, four T75 cell culture flasks were placed on the MuSIC in a 37 °C incubator (Figure S3E,F). For two hours, 1.8*g* gravitational load was then applied to the cell cultures, which corresponds to the hyper-*g*-phases during the PF. At the end of the run, the samples were fixed with RNAlater for RNA isolation. The 1*g* control samples were cultured in parallel and also fixed using RNAlater.

### 4.8. Vibration Experiment

T75 flasks containing subconfluent PC-3 cells were attached to the Vibraplex device (DLR, Cologne, Germany Figure S3G) and placed on the device in a 37 °C incubator in line with the protocol published earlier by Lützenberg et al. [51]. Vibrations between 0.2 and 14 Hz were transmitted via the Vibraplex using a connected loudspeaker for about 2 h. This procedure corresponds to frequencies generated during the PF. At the end of the two hours, the samples were fixed with RNAlater for RNA isolation. Static control samples were cultured and fixed with RNAlater in parallel.

### 4.9. RNA Sequencing

Extracted RNA was checked for quantity and quality as described in Section 4.3. Sufficient quality RNA was converted to libraries using the TruSeq™ RNA Library Prep Kit v2 (Illumina, San Diego, CA, USA) as directed by standard protocols. Libraries were sequenced on Illumina’s novaseq 6000 with paired-end 100 base pair reads and a sequencing depth of >20 milion pairs. For all sequencing data, reads were quality checked for read counts and quality values. The unaligned reads were mapped on the hg38 genome using STAR 2.6.1a [52] and the ENSEMBL v.99 [53] annotation. A maximum of ten mismatches and a multimapping to up to 10 different positions was allowed. Secondary alignments were filtered out.

### 4.10. Transcriptomic Analyses

We used the Bioconductor R package DESeq2 to capture differentially expressed genes (DEG). DESeq2 relies on a negative binomial generalized linear model (GLM) and uses the uniquely mapped RNAseq counts to an exon [54]. For integrated post hoc analysis for enrichments and networks of differentially expressed genes, we used the Database for Annotation, Visualization and Integrated Discovery (DAVID v. 6.8) [55] and the protein-to-protein interaction database STRING v. 11.5 [56], respectively. 

### 4.11. Statistical Analysis

The statistics were performed using the GraphPad Prism 7.01 software (GraphPad Software, Inc., San Diego, CA, USA) and the stats package of R 3.4.3. The sample and the related control were analyzed with the Mann–Whitney U-test at a significance level of *p* * < 0.05.

## 5. Conclusions

The objective behind our PF experiment was to identify early responses to the alternating gravitational stimulus and to put them into context with carcinogenesis and cancer progression in order to finally obtain new and fast expression-altering biomarkers for PC diagnosis. In general, we observed that the highly differential genes, which include some cytokines and especially chemokines, on the one hand provide a stable expression and are therefore robust for a diagnostic approach, and on the other hand are already related to the disease. Notably, the NGS analysis identified five cytokines (*CCL2*, *CXCL1*, *IL6*, *CXCL2*, *CCL20*), one zink finger protein (*TNFAIP3*), one glycoprotein (*ICAM1*) related to c-REL signaling, and the microRNA (*miR-221*) to be differentially regulated during PFs. Since these factors are relevant in cancer as well as inflammatory aspects, the presented findings may thus contribute to the development of improved diagnostic tools relevant to PC. CCL2/CCR2 have been associated with PC advancement, metastasis and relapse [57]. A similar action is known for IL6, which plays a key role in cancer progression [58]. In particular, non-coding RNAs and micro-RNAs have recently been increasingly used in cancer medicine as diagnostic, prognostic and therapeutic biomarkers [59,60,61,62] and cytokines are well established in cancer immunotherapy [63,64,65,66].

## Data Availability

Not applicable.

## Supplementary Materials

The following supporting information can be downloaded at: https://www.mdpi.com/article/10.3390/ijms23147876/s1.

## Abbreviations

PF	parabolic flight,
PC	prostate cancer
HNSCC	head and neck squamous cell carcinoma
HSCC	hypopharyngeal squamous cell carcinoma
PCA	principal component analysis
µ*g*	Microgravity
s-µ*g*	simulated microgravity
*p*	probability-value
P_adj_	adjusted probability-value
1P	one parabola
31P	31 parabolas
NGS	next generation sequencing
qPCR	quantitative real-time PCR
RPM	random positioning machine
HARV	high aspect rotating-wall vessel
PAM	PI3K/AKT/mTOR
