# Supplementary material for: In Prostate Cancer Cells Cytokines Are Early Responders to Gravitational Changes Occurring in Parabolic Flights
Source: Int J Mol Sci. 2022 Jul 17;23(14):7876. doi: 10.3390/ijms23147876 (PMC9319544; doi:10.3390/ijms23147876)
Supplement: Supplementary file 1 [file ijms-23-07876-s001.zip › ijms-1809794-supplementary.pdf]

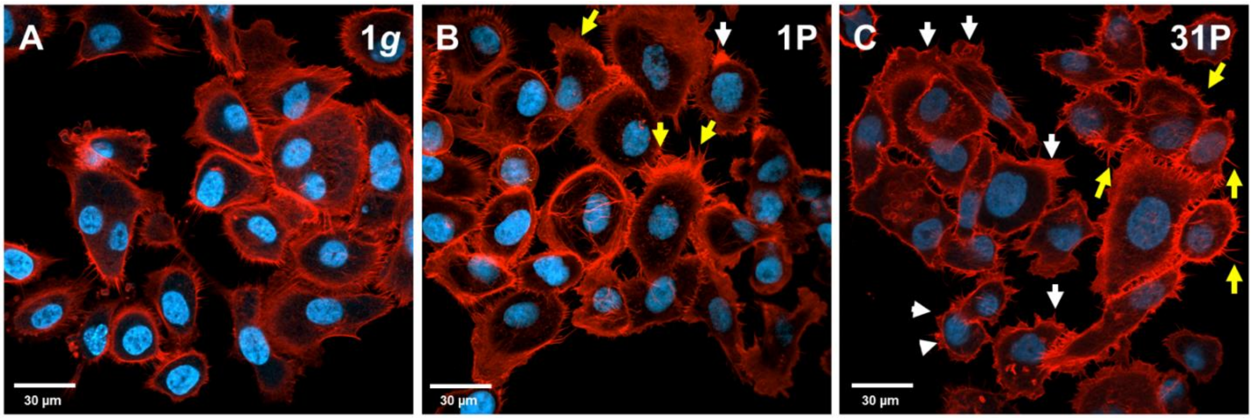

Figure S1: Impact of altered gravity on intracellular organization of F-actin: *Confocal laser scanning microscopy of TRITC-conjugated phalloidin stained (F-actin) samples.* ***A-C:*** *PC-3 cells cultivated under altered gravity conditions.* ***A:*** *Static 1g ground control.* ***B:*** *After the first parabola (1P).* ***C:*** *After the 31st parabola (31P). The white arrows show psudopoida and lamellipodia and yellow arrows indicate stress fibers. Scale bars represent 30 µm.*

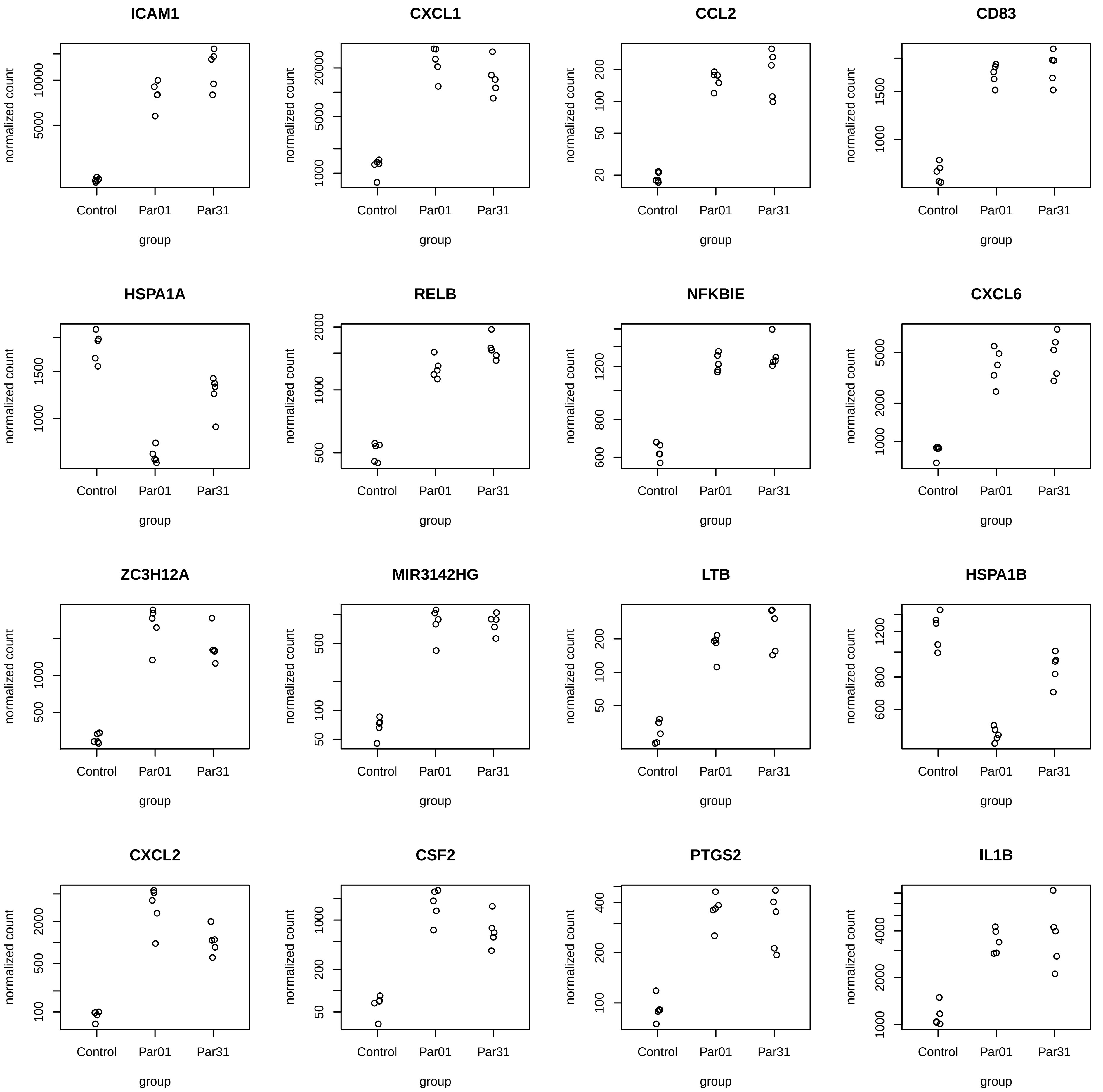
ICAM1
normalized count
5000
10000
Control
Par01
Par31
group
CXCL1
normalized count
1000
5000
20000
Control
Par01
Par31
group
CCL2
normalized count
20
50
100
200
Control
Par01
Par31
group
CD83
normalized count
1000
1500
Control
Par01
Par31
group
HSPA1A
normalized count
1000
1500
Control
Par01
Par31
group
RELB
normalized count
500
1000
2000
Control
Par01
Par31
group
NFKBIE
normalized count
600
800
1200
Control
Par01
Par31
group
CXCL6
normalized count
1000
2000
5000
Control
Par01
Par31
group
ZC3H12A
normalized count
500
1000
Control
Par01
Par31
group
MIR3142HG
normalized count
50
100
500
Control
Par01
Par31
group
LTB
normalized count
50
100
200
Control
Par01
Par31
group
HSPA1B
normalized count
600
800
1200
Control
Par01
Par31
group
CXCL2
normalized count
100
500
2000
Control
Par01
Par31
group
CSF2
normalized count
50
200
1000
Control
Par01
Par31
group
PTGS2
normalized count
100
200
400
Control
Par01
Par31
group
IL1B
normalized count
1000
2000
4000
Control
Par01
Par31
group

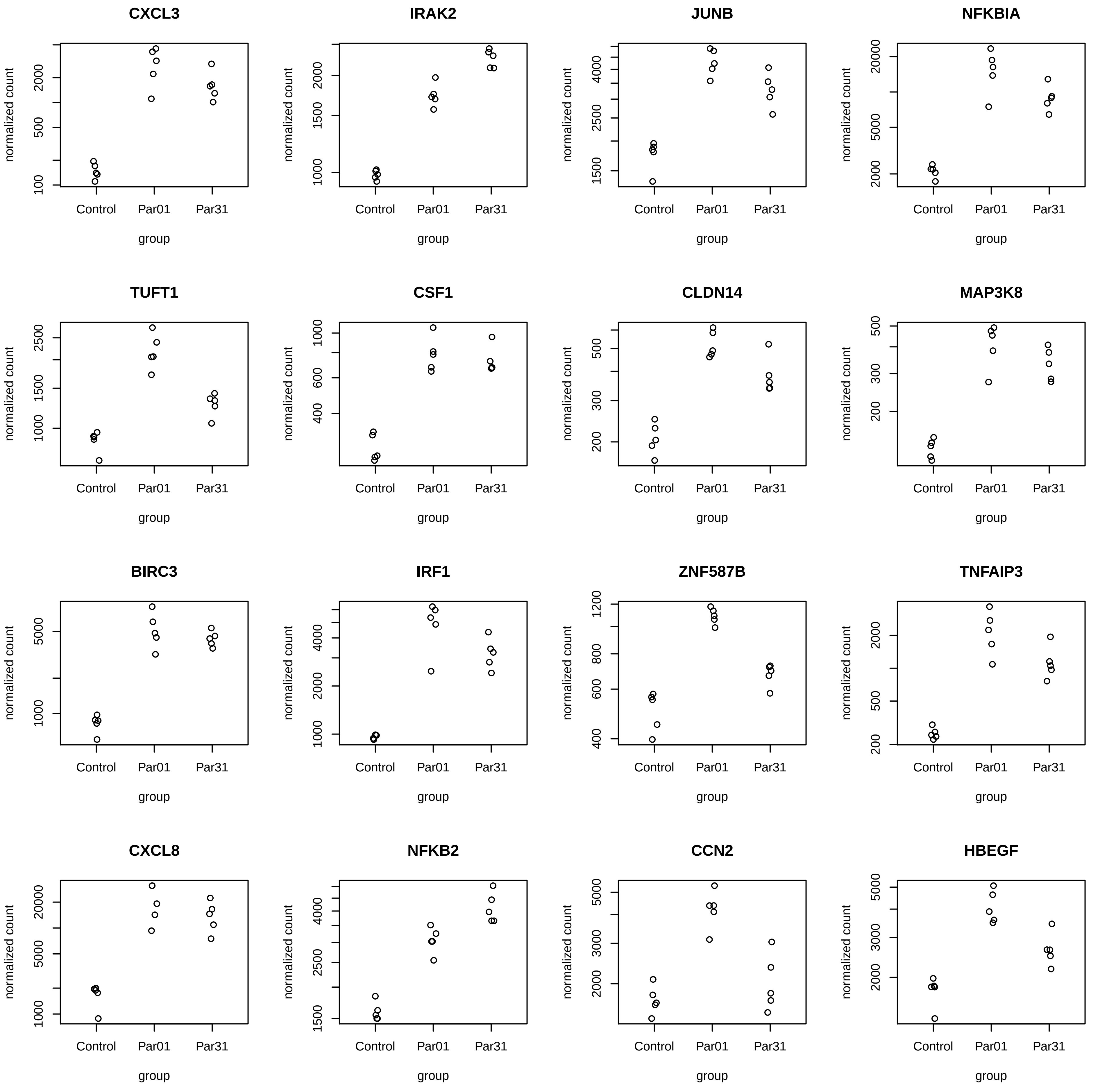
CXCL3
normalized count
100
500
2000
Control
Par01
Par31
group
IRAK2
normalized count
1000
1500
2000
Control
Par01
Par31
group
JUNB
normalized count
1500
2500
4000
Control
Par01
Par31
group
NFKBIA
normalized count
2000
5000
20000
Control
Par01
Par31
group
TUFT1
normalized count
1000
1500
2500
Control
Par01
Par31
group
CSF1
normalized count
400
600
1000
Control
Par01
Par31
group
CLDN14
normalized count
200
300
500
Control
Par01
Par31
group
MAP3K8
normalized count
200
300
500
Control
Par01
Par31
group
BIRC3
normalized count
1000
5000
Control
Par01
Par31
group
IRF1
normalized count
1000
2000
4000
Control
Par01
Par31
group
ZNF587B
normalized count
400
600
800
1200
Control
Par01
Par31
group
TNFAIP3
normalized count
200
500
2000
Control
Par01
Par31
group
CXCL8
normalized count
1000
5000
20000
Control
Par01
Par31
group
NFKB2
normalized count
1500
2500
4000
Control
Par01
Par31
group
CCN2
normalized count
2000
3000
5000
Control
Par01
Par31
group
HBEGF
normalized count
2000
3000
5000
Control
Par01
Par31
group

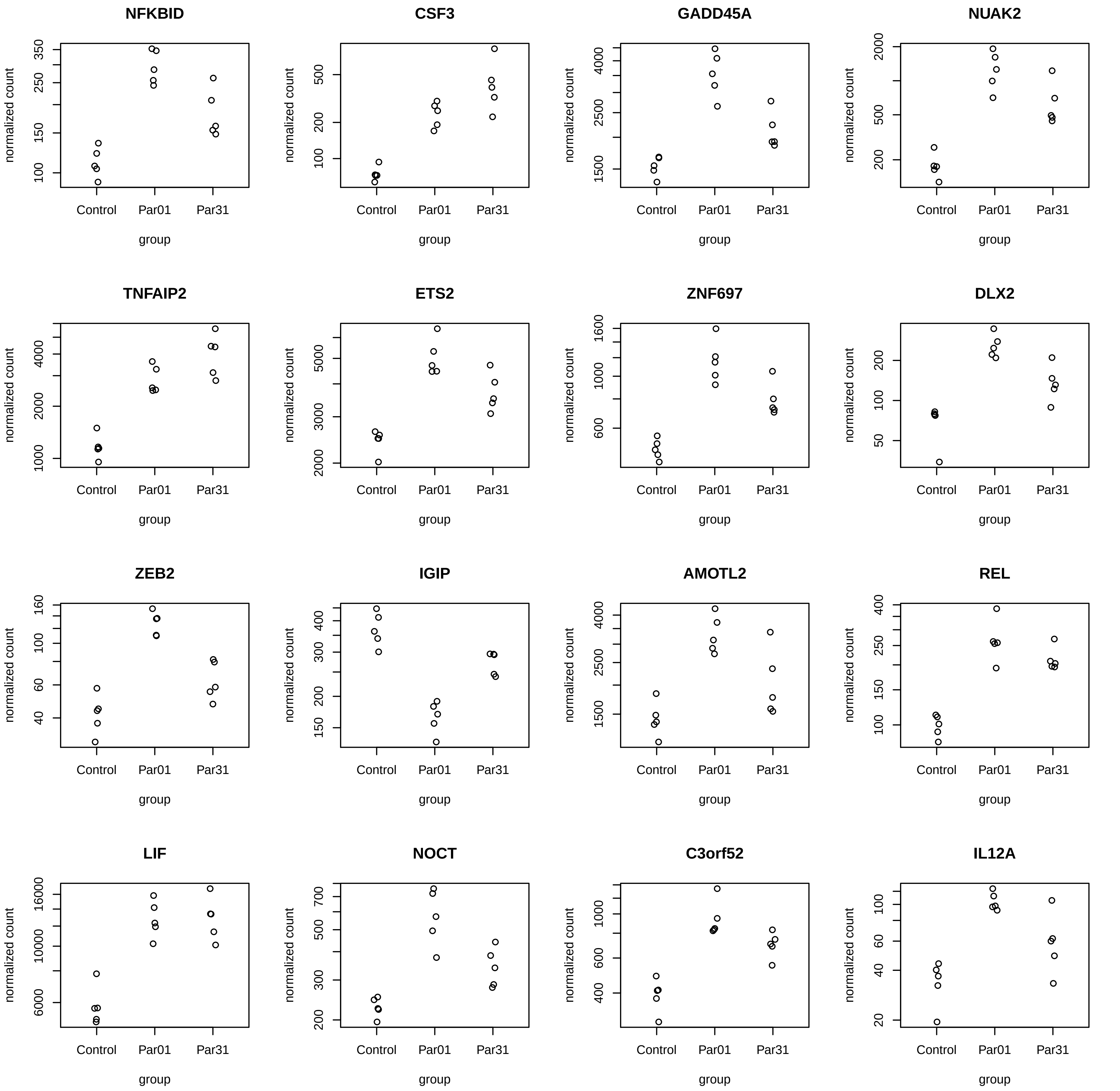
NFKBID
normalized count
100
150
250
350
Control
Par01
Par31
group
CSF3
normalized count
100
200
500
Control
Par01
Par31
group
GADD45A
normalized count
1500
2500
4000
Control
Par01
Par31
group
NUAK2
normalized count
200
500
2000
Control
Par01
Par31
group
TNFAIP2
normalized count
1000
2000
4000
Control
Par01
Par31
group
ETS2
normalized count
2000
3000
5000
Control
Par01
Par31
group
ZNF697
normalized count
600
1000
1600
Control
Par01
Par31
group
DLX2
normalized count
50
100
200
Control
Par01
Par31
group
ZEB2
normalized count
40
60
100
160
Control
Par01
Par31
group
IGIP
normalized count
150
200
300
400
Control
Par01
Par31
group
AMOTL2
normalized count
1500
2500
4000
Control
Par01
Par31
group
REL
normalized count
100
150
250
400
Control
Par01
Par31
group
LIF
normalized count
6000
10000
16000
Control
Par01
Par31
group
NOCT
normalized count
200
300
500
700
Control
Par01
Par31
group
C3orf52
normalized count
400
600
1000
Control
Par01
Par31
group
IL12A
normalized count
20
40
60
100
Control
Par01
Par31
group

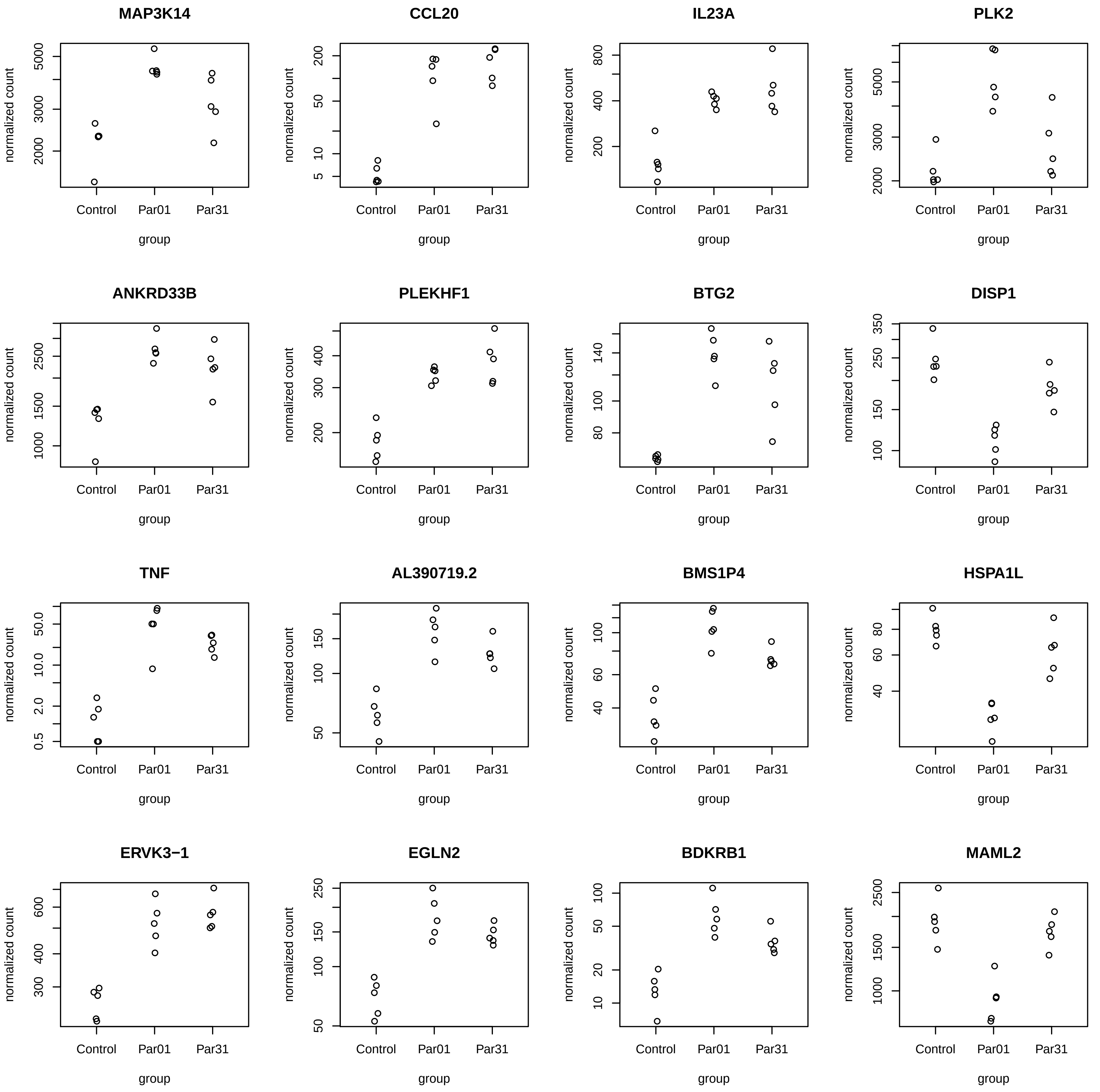
MAP3K14
normalized count
2000 3000 5000
Control Par01 Par31
group
CCL20
normalized count
5 10 50 200
Control Par01 Par31
group
IL23A
normalized count
200 400 800
Control Par01 Par31
group
PLK2
normalized count
2000 3000 5000
Control Par01 Par31
group
ANKRD33B
normalized count
1000 1500 2500
Control Par01 Par31
group
PLEKHF1
normalized count
200 300 400
Control Par01 Par31
group
BTG2
normalized count
80 100 140
Control Par01 Par31
group
DISP1
normalized count
100 150 250 350
Control Par01 Par31
group
TNF
normalized count
0.5 2.0 10.0 50.0
Control Par01 Par31
group
AL390719.2
normalized count
50 100 150
Control Par01 Par31
group
BMS1P4
normalized count
40 60 100
Control Par01 Par31
group
HSPA1L
normalized count
40 60 80
Control Par01 Par31
group
ERVK3−1
normalized count
300 400 600
Control Par01 Par31
group
EGLN2
normalized count
50 100 150 250
Control Par01 Par31
group
BDKRB1
normalized count
10 20 50 100
Control Par01 Par31
group
MAML2
normalized count
1000 1500 2500
Control Par01 Par31
group

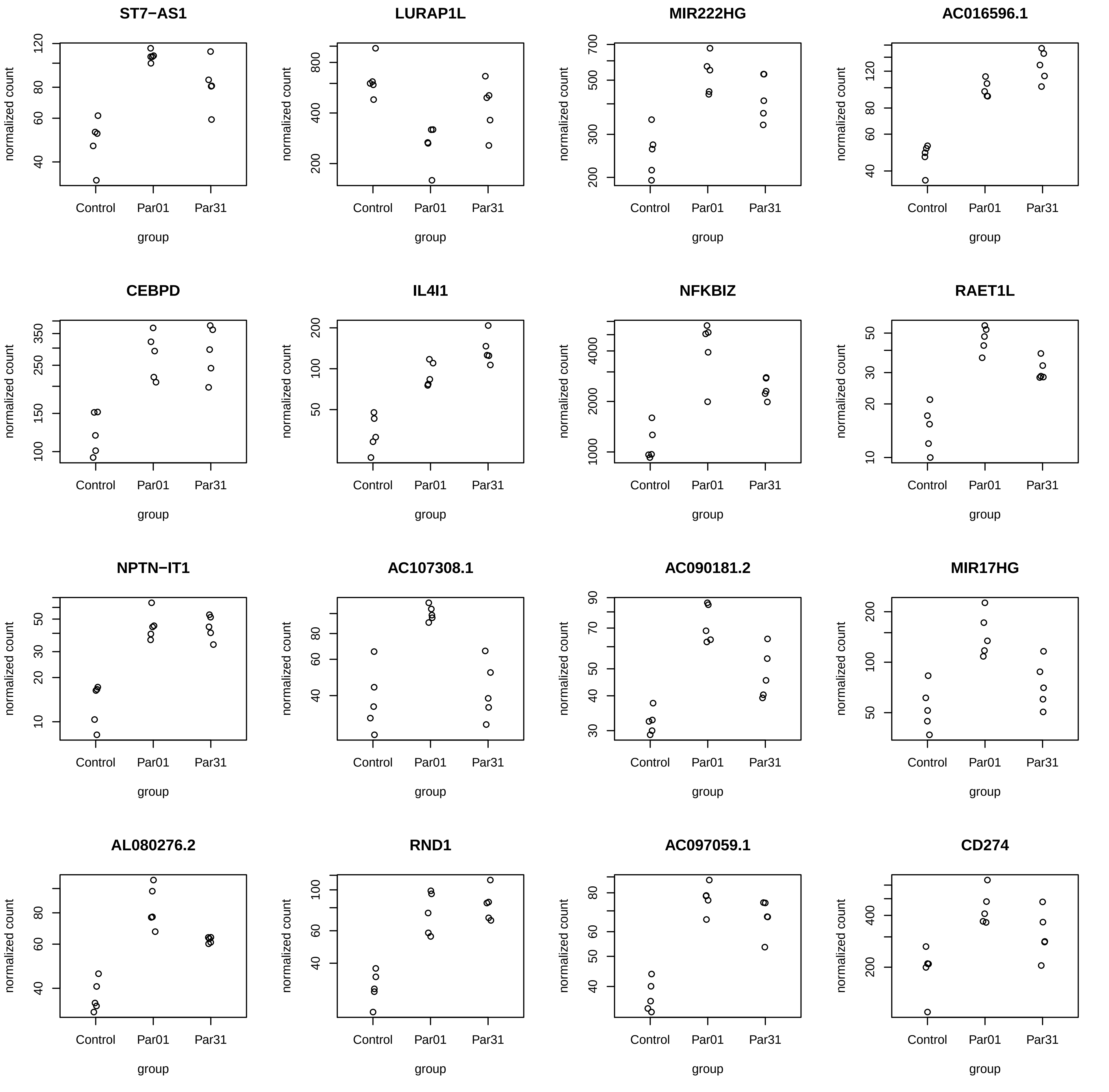
ST7−AS1
normalized count
40
60
80
120
Control
Par01
Par31
group
LURAP1L
normalized count
200
400
800
Control
Par01
Par31
group
MIR222HG
normalized count
200
300
500
700
Control
Par01
Par31
group
AC016596.1
normalized count
40
60
80
120
Control
Par01
Par31
group
CEBPD
normalized count
100
150
250
350
Control
Par01
Par31
group
IL4I1
normalized count
50
100
200
Control
Par01
Par31
group
NFKBIZ
normalized count
1000
2000
4000
Control
Par01
Par31
group
RAET1L
normalized count
10
20
30
50
Control
Par01
Par31
group
NPTN−IT1
normalized count
10
20
30
50
Control
Par01
Par31
group
AC107308.1
normalized count
40
60
80
Control
Par01
Par31
group
AC090181.2
normalized count
30
40
50
70
90
Control
Par01
Par31
group
MIR17HG
normalized count
50
100
200
Control
Par01
Par31
group
AL080276.2
normalized count
40
60
80
Control
Par01
Par31
group
RND1
normalized count
40
60
100
Control
Par01
Par31
group
AC097059.1
normalized count
40
50
60
80
Control
Par01
Par31
group
CD274
normalized count
200
400
Control
Par01
Par31
group

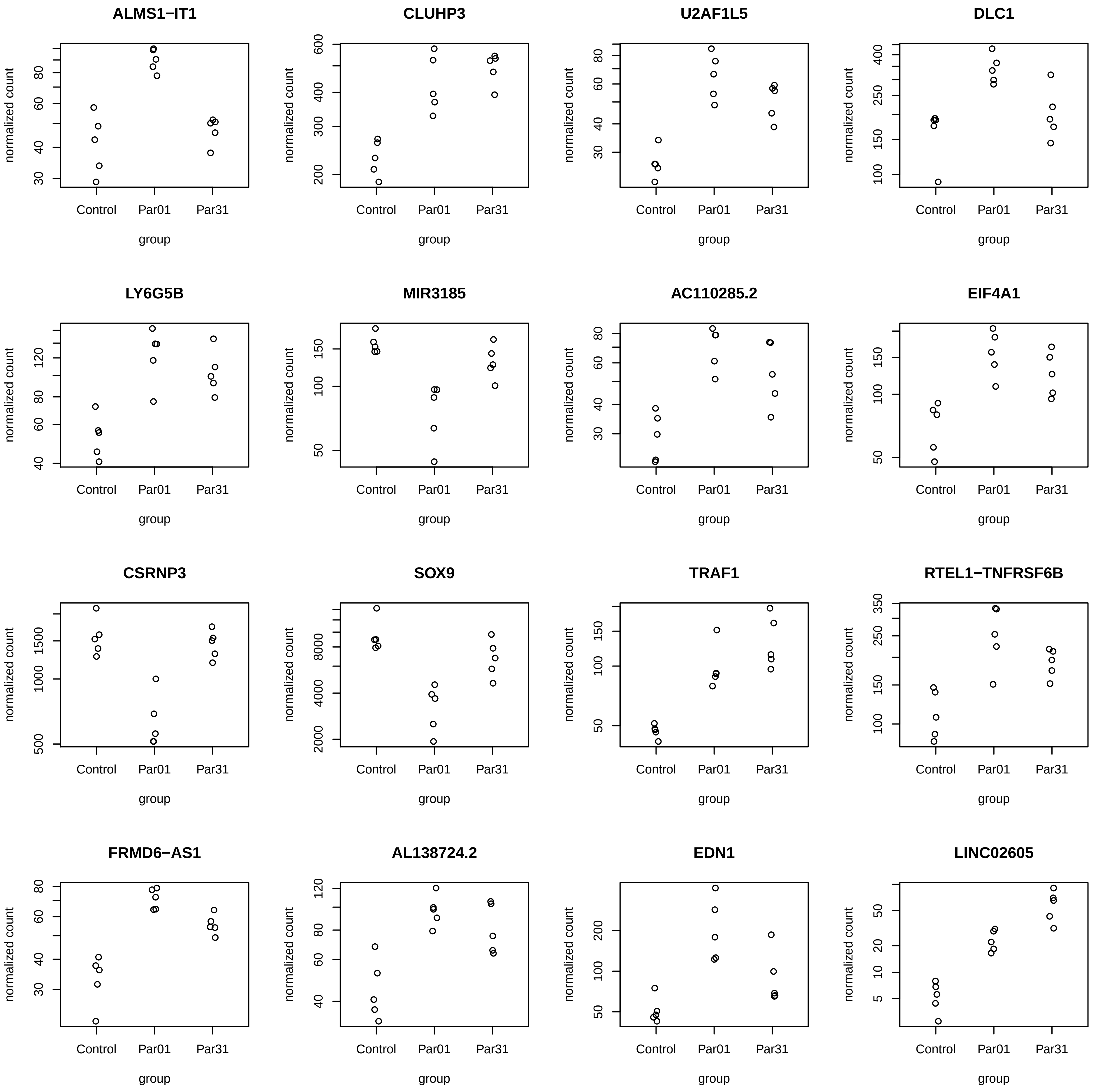

ALMS1−IT1
CLUHP3
U2AF1L5
DLC1
LY6G5B
MIR3185
AC110285.2
EIF4A1
CSRNP3
SOX9
TRAF1
RTEL1−TNFRSF6B
FRMD6−AS1
AL138724.2
EDN1
LINC02605
normalized count
Control
Par01
Par31
group

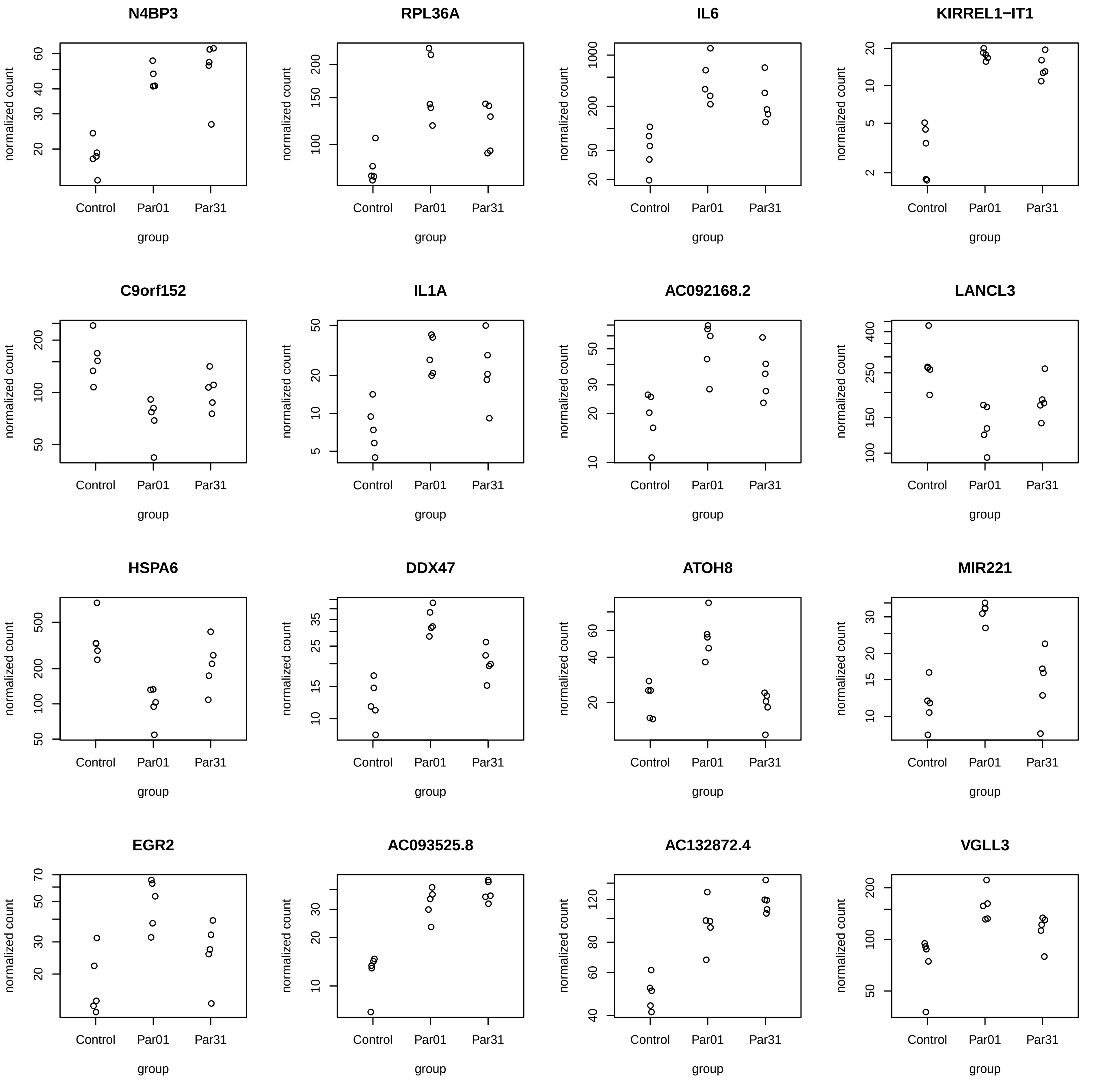
N4BP3
RPL36A
IL6
KIRREL1−IT1
C9orf152
IL1A
AC092168.2
LANCL3
HSPA6
DDX47
ATOH8
MIR221
EGR2
AC093525.8
AC132872.4
VGLL3
normalized count
group
Control
Par01
Par31
N4BP3: 20 30 40 60
RPL36A: 100 150 200
IL6: 20 50 200 1000
KIRREL1−IT1: 2 5 10 20
C9orf152: 50 100 200
IL1A: 5 10 20 50
AC092168.2: 10 20 30 50
LANCL3: 100 150 250 400
HSPA6: 50 100 200 500
DDX47: 10 15 25 35
ATOH8: 20 40 60
MIR221: 10 15 20 30
EGR2: 20 30 50 70
AC093525.8: 10 20 30
AC132872.4: 40 60 80 120
VGLL3: 50 100 200

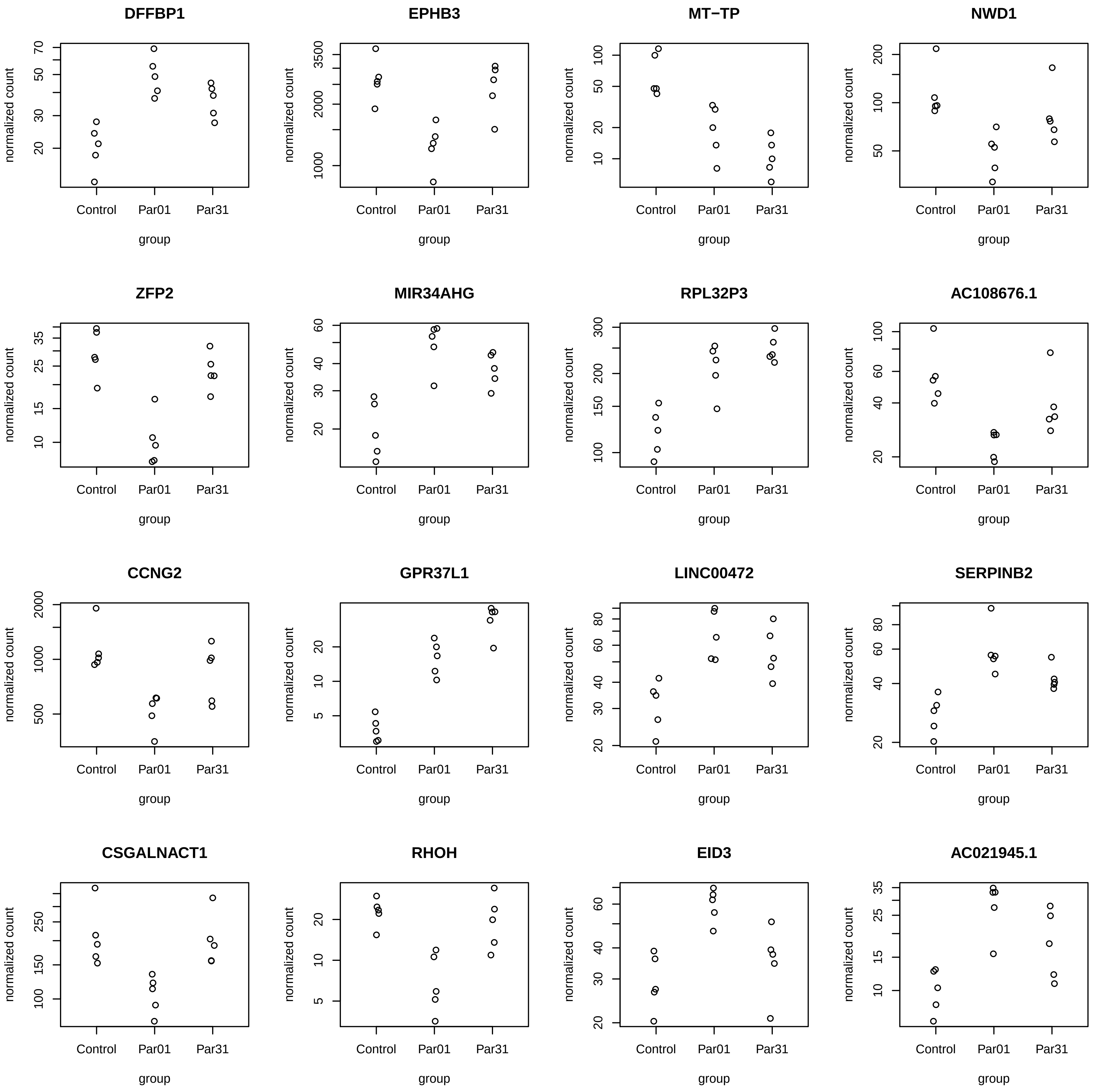

DFFBP1
EPHB3
MT−TP
NWD1
ZFP2
MIR34AHG
RPL32P3
AC108676.1
CCNG2
GPR37L1
LINC00472
SERPINB2
CSGALNACT1
RHOH
EID3
AC021945.1
normalized count
Control
Par01
Par31
group

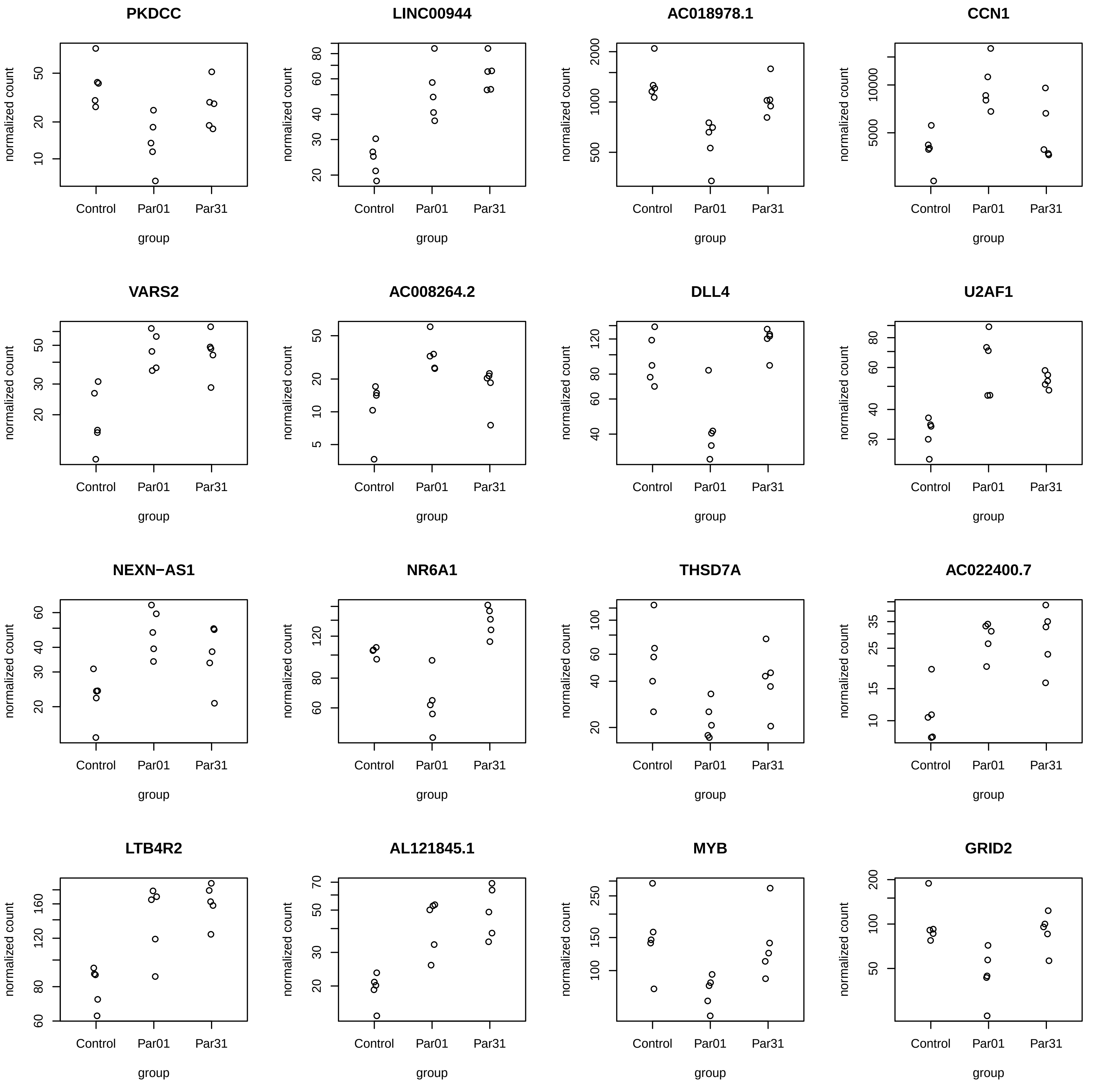

PKDCC
LINC00944
AC018978.1
CCN1
VARS2
AC008264.2
DLL4
U2AF1
NEXN−AS1
NR6A1
THSD7A
AC022400.7
LTB4R2
AL121845.1
MYB
GRID2
normalized count
group
Control
Par01
Par31

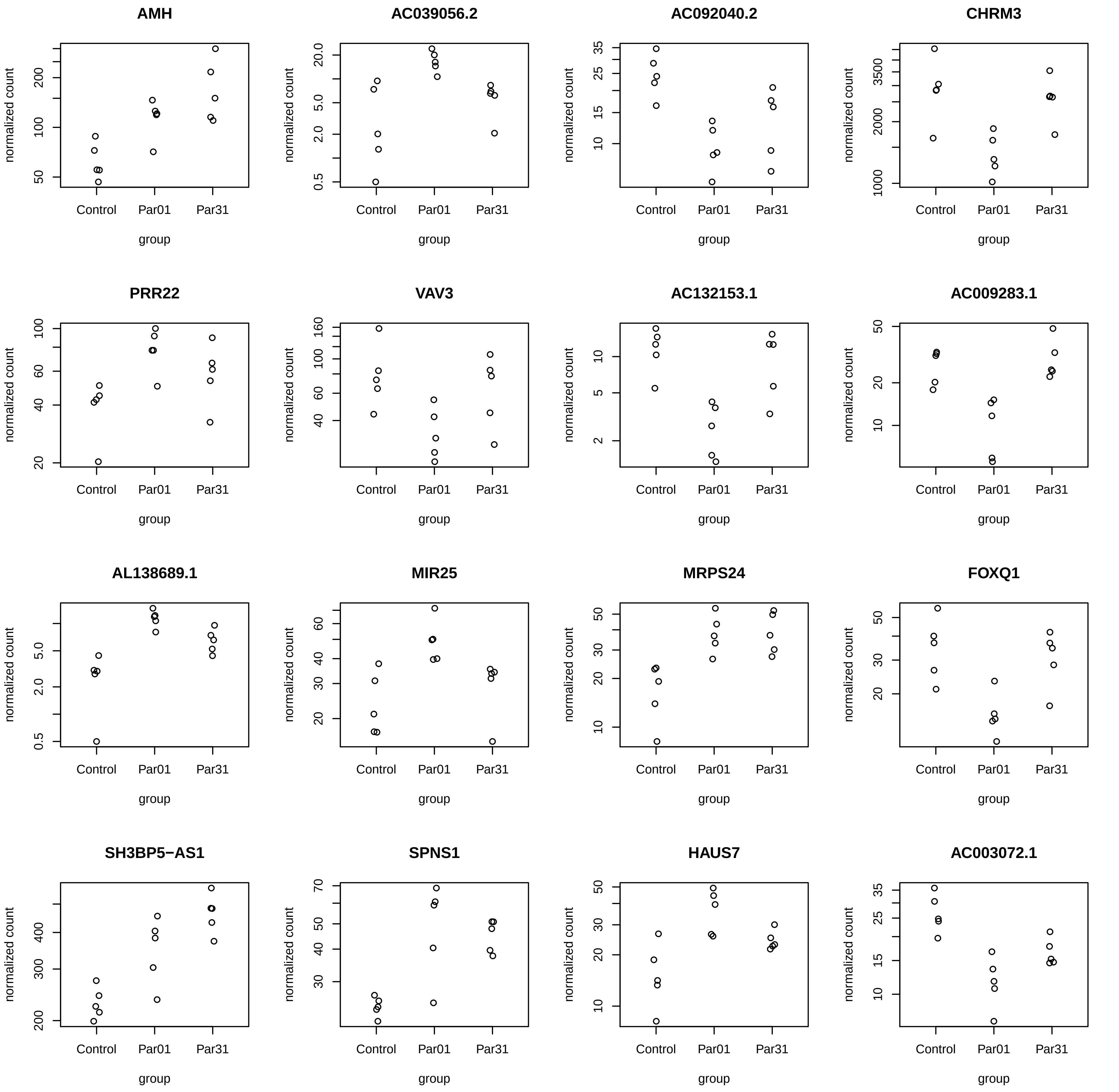

AMH
normalized count
50
100
200
Control
Par01
Par31
group
AC039056.2
normalized count
0.5
2.0
5.0
20.0
Control
Par01
Par31
group
AC092040.2
normalized count
10
15
25
35
Control
Par01
Par31
group
CHRM3
normalized count
1000
2000
3500
Control
Par01
Par31
group
PRR22
normalized count
20
40
60
100
Control
Par01
Par31
group
VAV3
normalized count
40
60
100
160
Control
Par01
Par31
group
AC132153.1
normalized count
2
5
10
Control
Par01
Par31
group
AC009283.1
normalized count
10
20
50
Control
Par01
Par31
group
AL138689.1
normalized count
0.5
2.0
5.0
Control
Par01
Par31
group
MIR25
normalized count
20
30
40
60
Control
Par01
Par31
group
MRPS24
normalized count
10
20
30
50
Control
Par01
Par31
group
FOXQ1
normalized count
20
30
50
Control
Par01
Par31
group
SH3BP5−AS1
normalized count
200
300
400
Control
Par01
Par31
group
SPNS1
normalized count
30
40
50
70
Control
Par01
Par31
group
HAUS7
normalized count
10
20
30
50
Control
Par01
Par31
group
AC003072.1
normalized count
10
15
25
35
Control
Par01
Par31
group

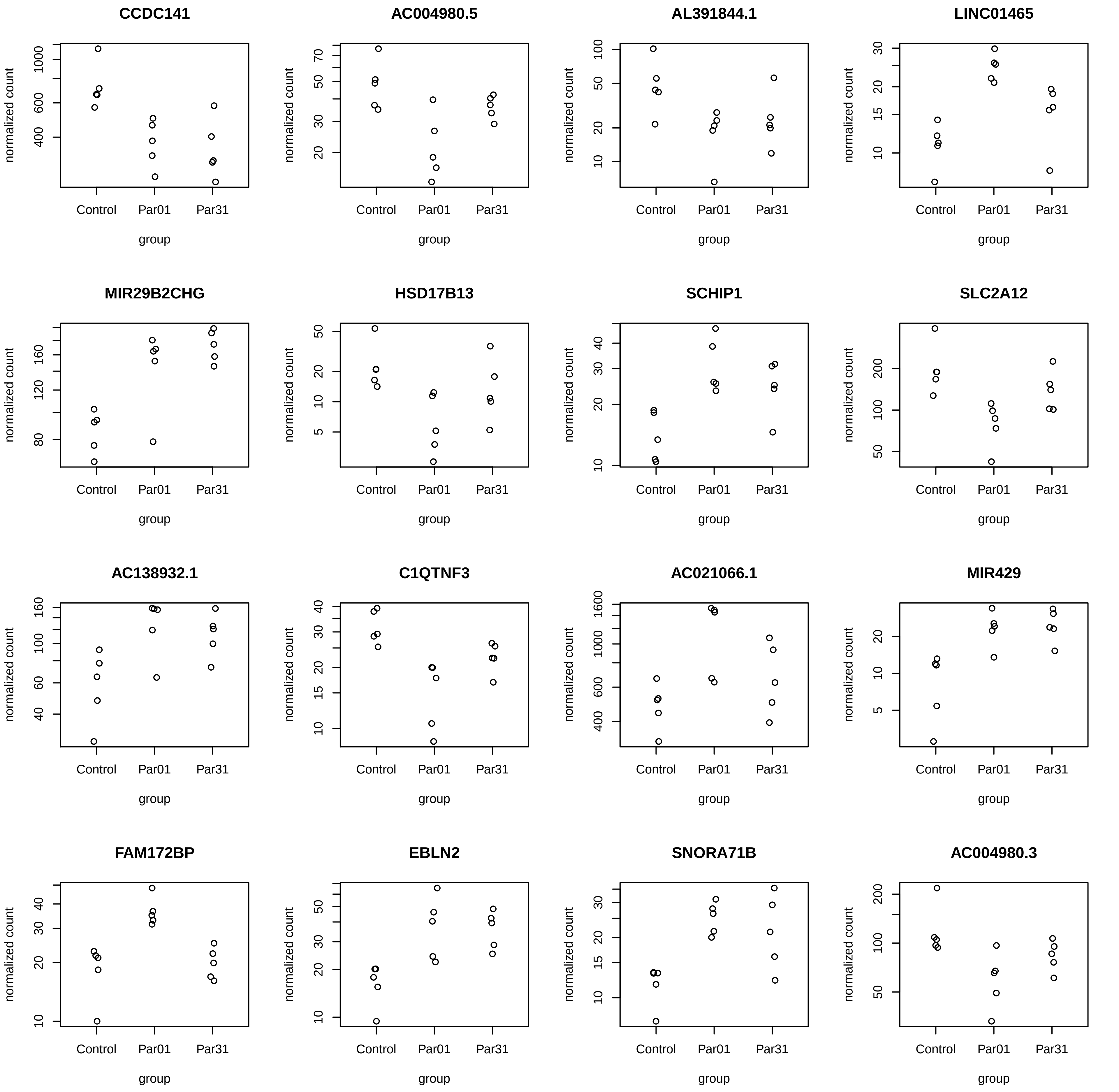
CCDC141
400
600
1000
normalized count
Control
Par01
Par31
group
AC004980.5
20
30
50
70
normalized count
Control
Par01
Par31
group
AL391844.1
10
20
50
100
normalized count
Control
Par01
Par31
group
LINC01465
10
15
20
30
normalized count
Control
Par01
Par31
group
MIR29B2CHG
80
120
160
normalized count
Control
Par01
Par31
group
HSD17B13
5
10
20
50
normalized count
Control
Par01
Par31
group
SCHIP1
10
20
30
40
normalized count
Control
Par01
Par31
group
SLC2A12
50
100
200
normalized count
Control
Par01
Par31
group
AC138932.1
40
60
100
160
normalized count
Control
Par01
Par31
group
C1QTNF3
10
15
20
30
40
normalized count
Control
Par01
Par31
group
AC021066.1
400
600
1000
1600
normalized count
Control
Par01
Par31
group
MIR429
5
10
20
normalized count
Control
Par01
Par31
group
FAM172BP
10
20
30
40
normalized count
Control
Par01
Par31
group
EBLN2
10
20
30
50
normalized count
Control
Par01
Par31
group
SNORA71B
10
15
20
30
normalized count
Control
Par01
Par31
group
AC004980.3
50
100
200
normalized count
Control
Par01
Par31
group

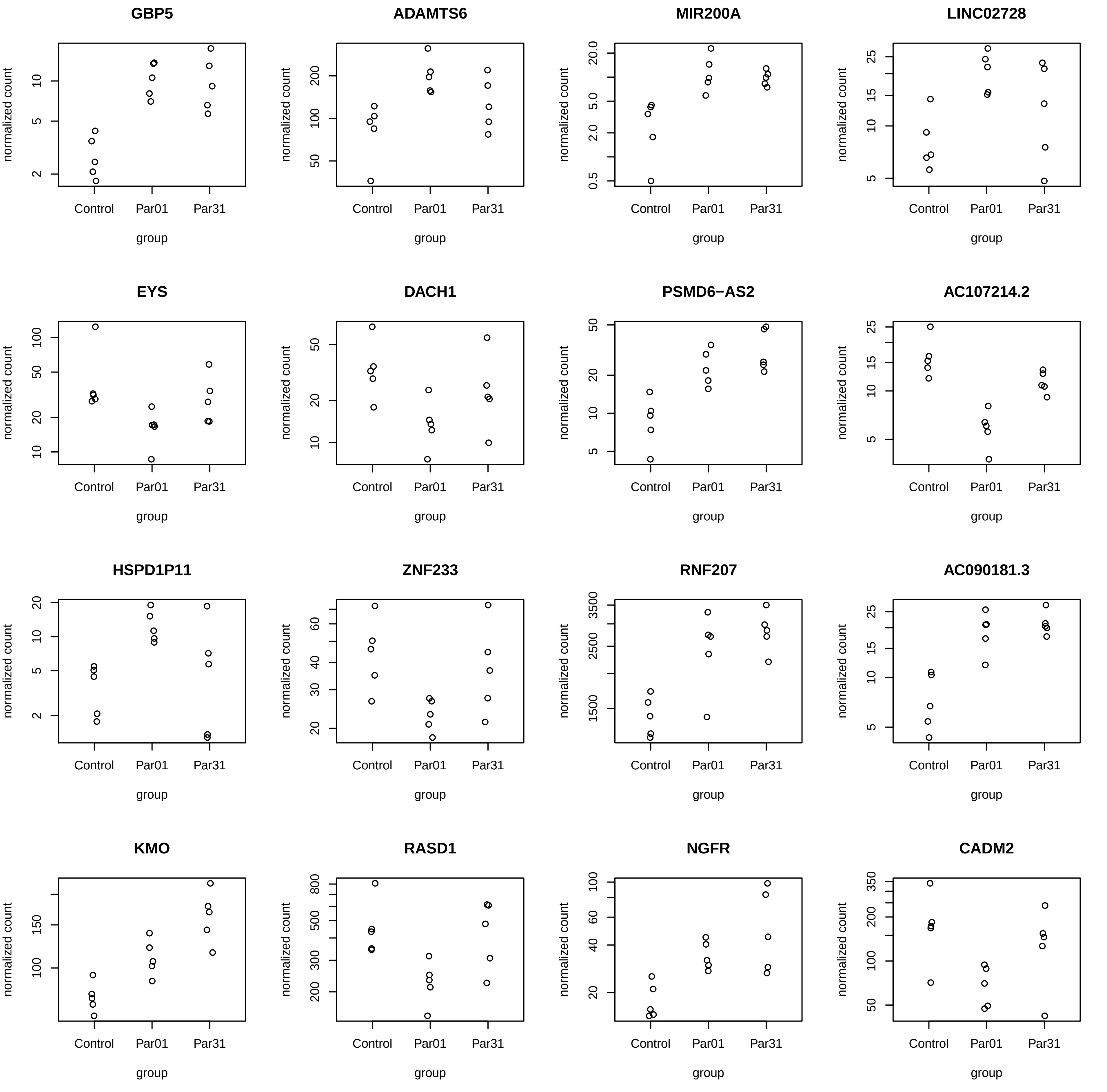

GBP5
ADAMTS6
MIR200A
LINC02728
EYS
DACH1
PSMD6−AS2
AC107214.2
HSPD1P11
ZNF233
RNF207
AC090181.3
KMO
RASD1
NGFR
CADM2
normalized count
group
Control
Par01
Par31

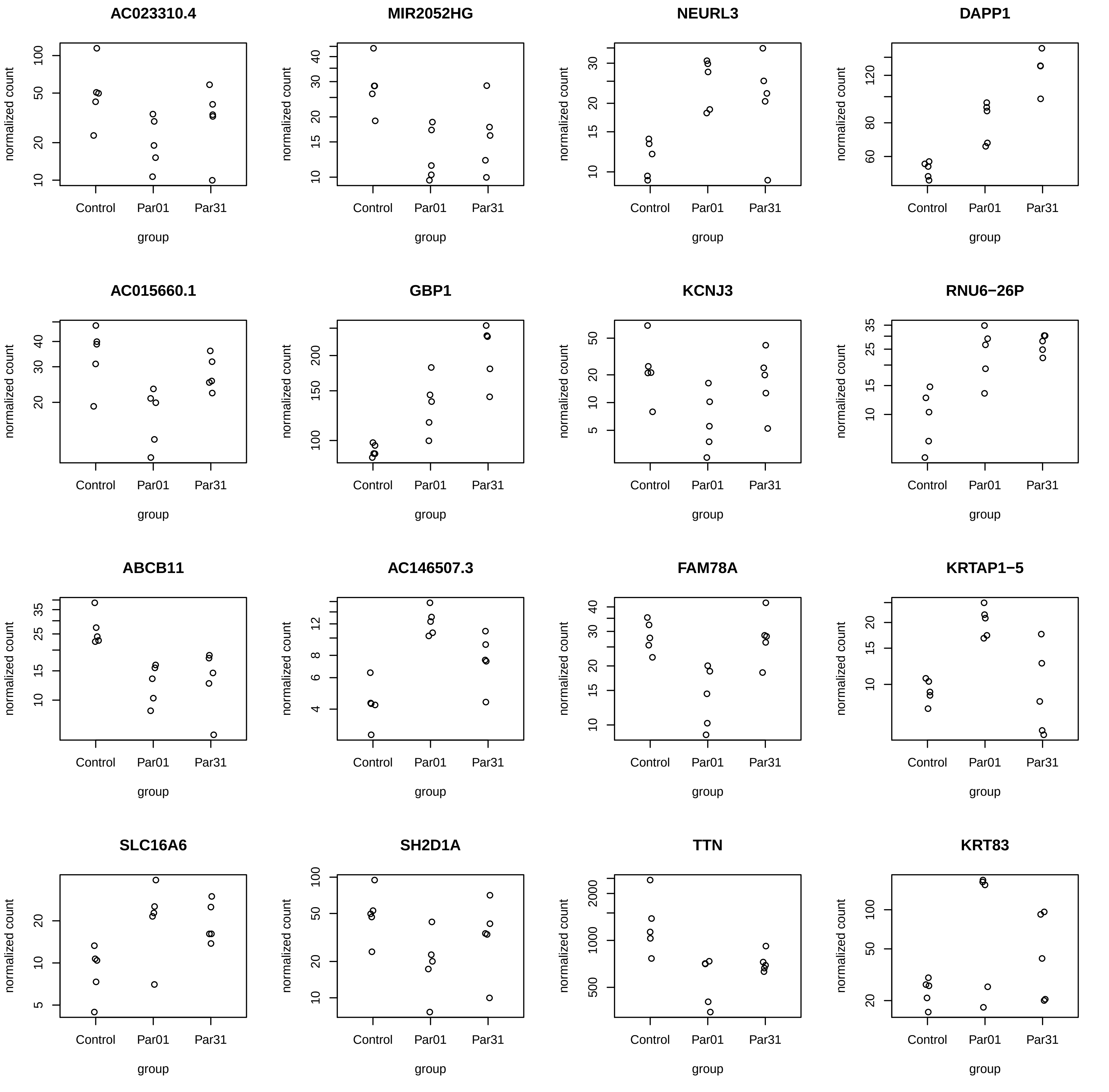
AC023310.4
MIR2052HG
NEURL3
DAPP1
AC015660.1
GBP1
KCNJ3
RNU6−26P
ABCB11
AC146507.3
FAM78A
KRTAP1−5
SLC16A6
SH2D1A
TTN
KRT83
normalized count
Control
Par01
Par31
group

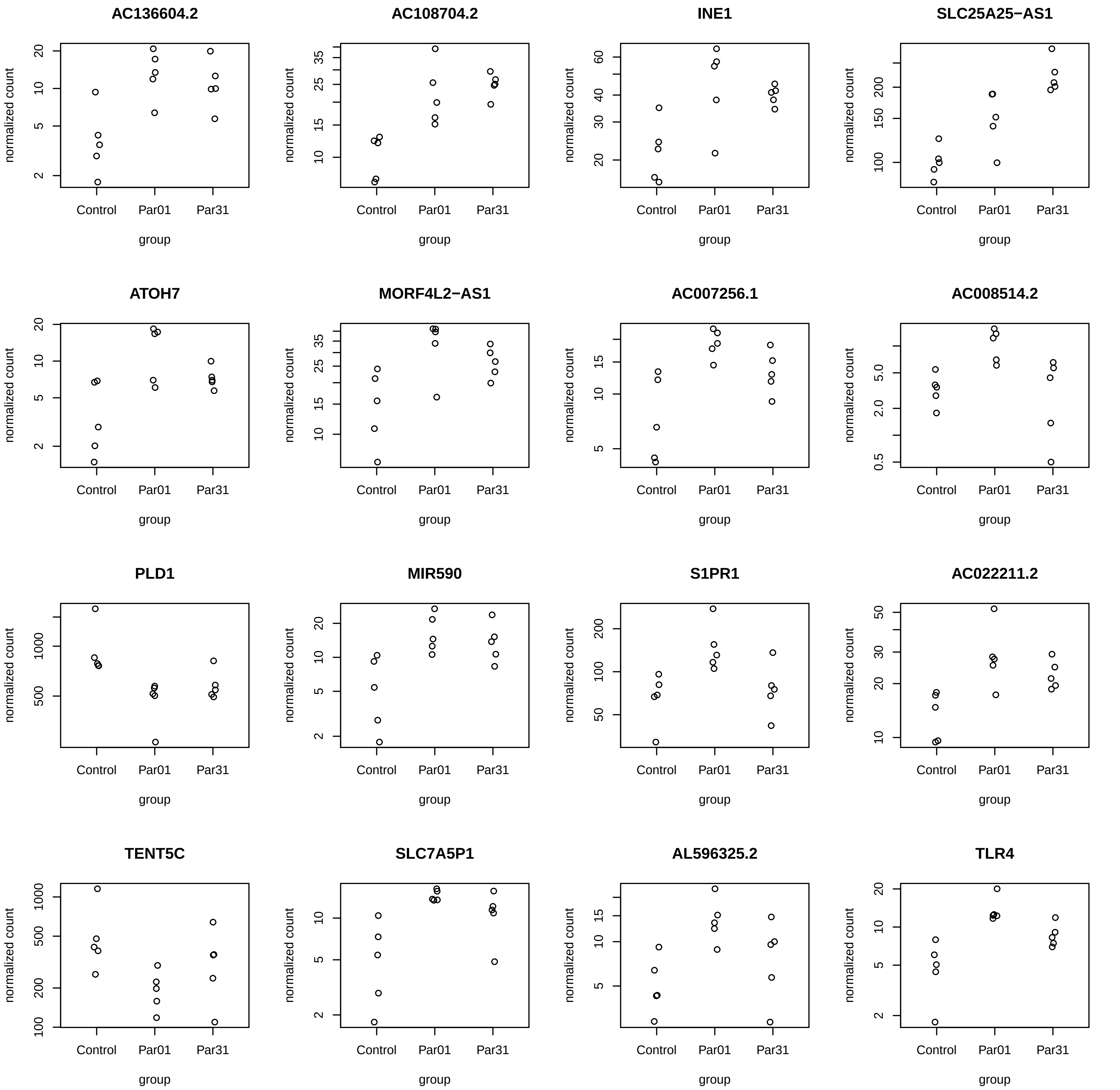
AC136604.2
normalized count
2 5 10 20
Control Par01 Par31
group
AC108704.2
normalized count
10 15 25 35
Control Par01 Par31
group
INE1
normalized count
20 30 40 60
Control Par01 Par31
group
SLC25A25−AS1
normalized count
100 150 200
Control Par01 Par31
group
ATOH7
normalized count
2 5 10 20
Control Par01 Par31
group
MORF4L2−AS1
normalized count
10 15 25 35
Control Par01 Par31
group
AC007256.1
normalized count
5 10 15
Control Par01 Par31
group
AC008514.2
normalized count
0.5 2.0 5.0
Control Par01 Par31
group
PLD1
normalized count
500 1000
Control Par01 Par31
group
MIR590
normalized count
2 5 10 20
Control Par01 Par31
group
S1PR1
normalized count
50 100 200
Control Par01 Par31
group
AC022211.2
normalized count
10 20 30 50
Control Par01 Par31
group
TENT5C
normalized count
100 200 500 1000
Control Par01 Par31
group
SLC7A5P1
normalized count
2 5 10
Control Par01 Par31
group
AL596325.2
normalized count
5 10 15
Control Par01 Par31
group
TLR4
normalized count
2 5 10 20
Control Par01 Par31
group

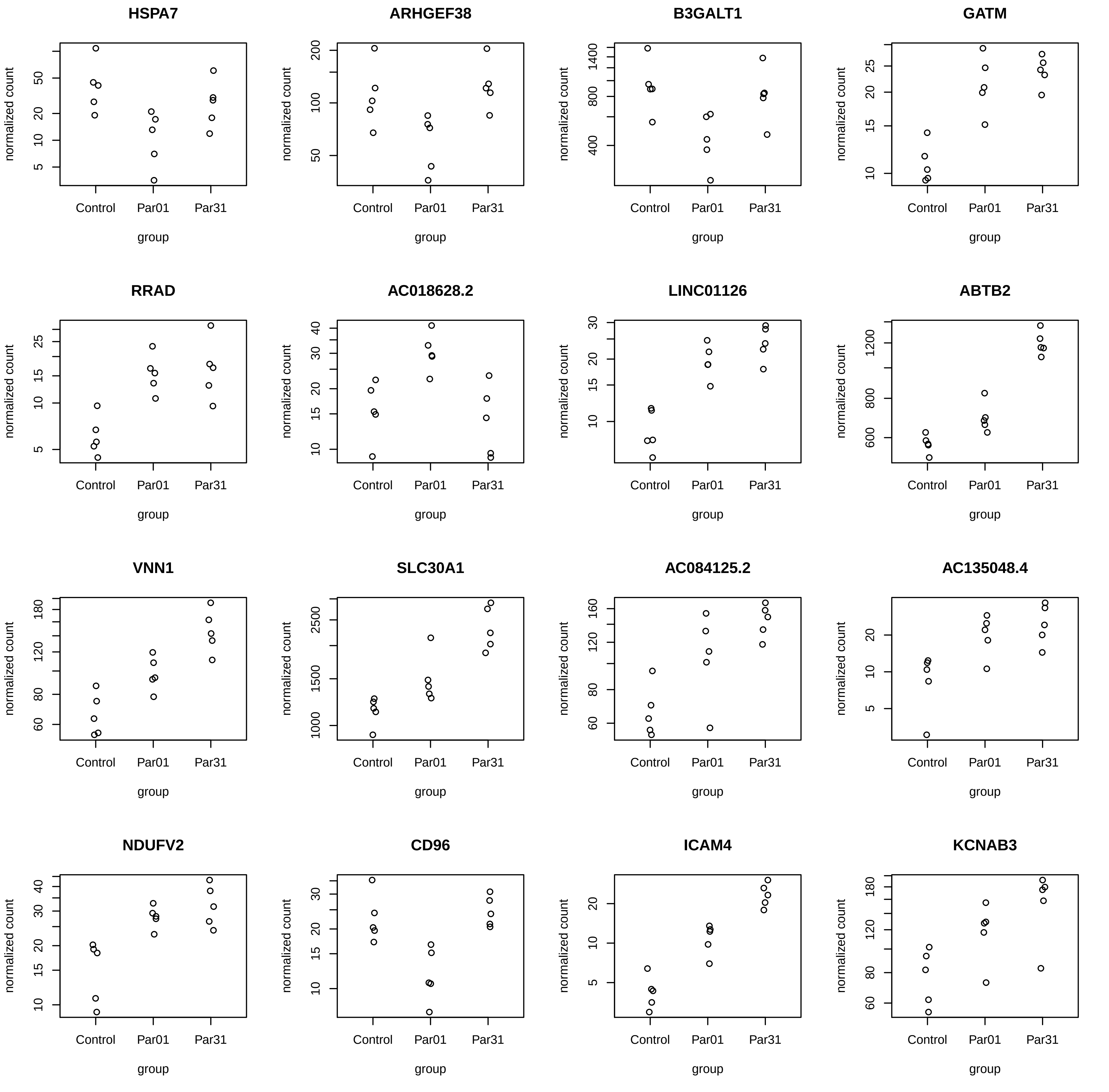
HSPA7
ARHGEF38
B3GALT1
GATM
RRAD
AC018628.2
LINC01126
ABTB2
VNN1
SLC30A1
AC084125.2
AC135048.4
NDUFV2
CD96
ICAM4
KCNAB3
normalized count
group
Control
Par01
Par31
5 10 20 50
50 100 200
400 800 1400
10 15 20 25
5 10 15 25
10 15 20 30 40
10 15 20 30
600 800 1200
60 80 120 180
1000 1500 2500
60 80 120 160
5 10 20
10 15 20 30 40
10 15 20 30
5 10 20
60 80 120 180

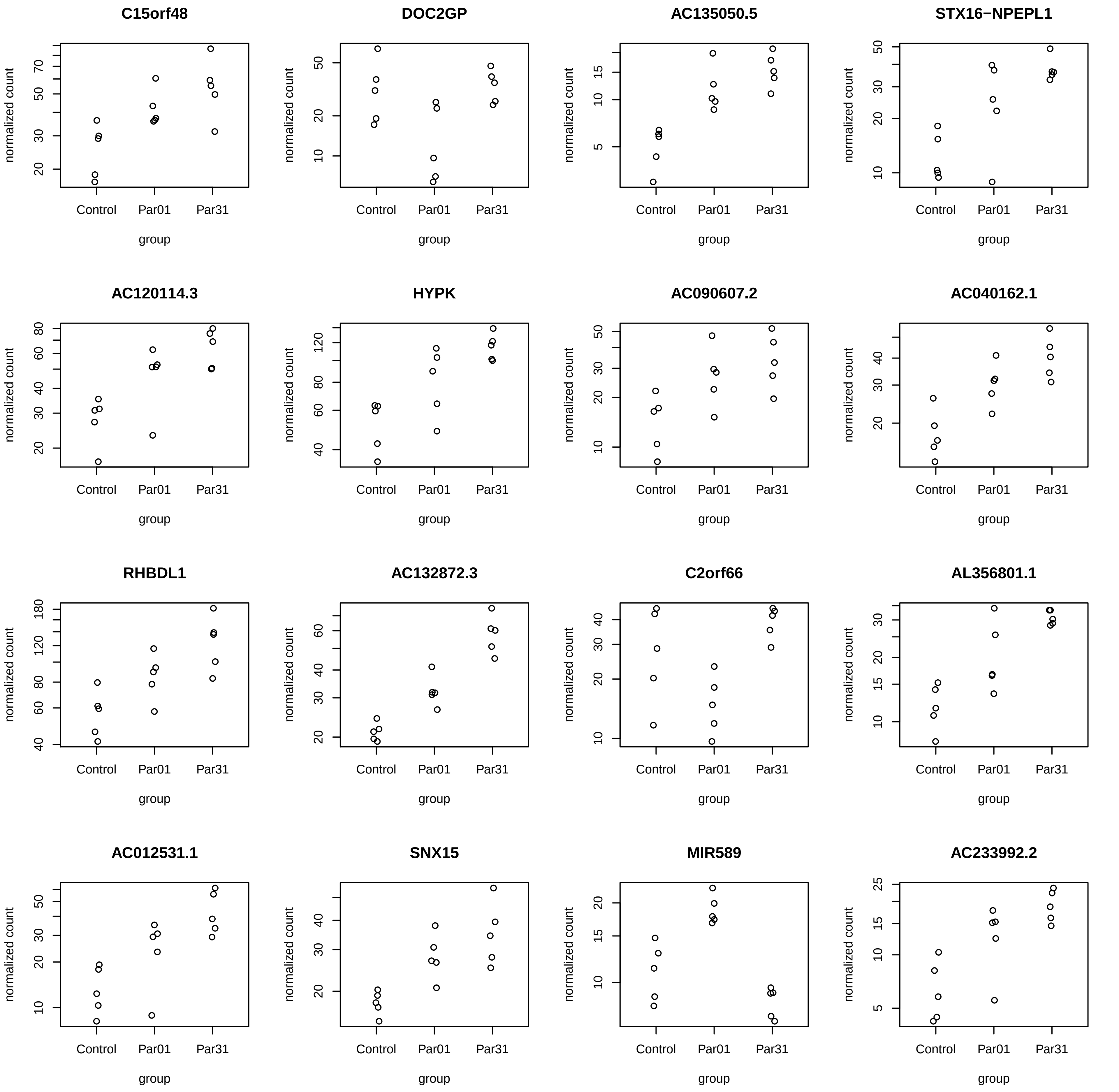

C15orf48
20
30
50
70
DOC2GP
10
20
50
AC135050.5
5
10
15
STX16−NPEPL1
10
20
30
50
AC120114.3
20
30
40
60
80
HYPK
40
60
80
120
AC090607.2
10
20
30
50
AC040162.1
20
30
40
RHBDL1
40
60
80
120
180
AC132872.3
20
30
40
60
C2orf66
10
20
30
40
AL356801.1
10
15
20
30
AC012531.1
10
20
30
50
SNX15
20
30
40
MIR589
10
15
20
AC233992.2
5
10
15
25
normalized count
Control
Par01
Par31
group

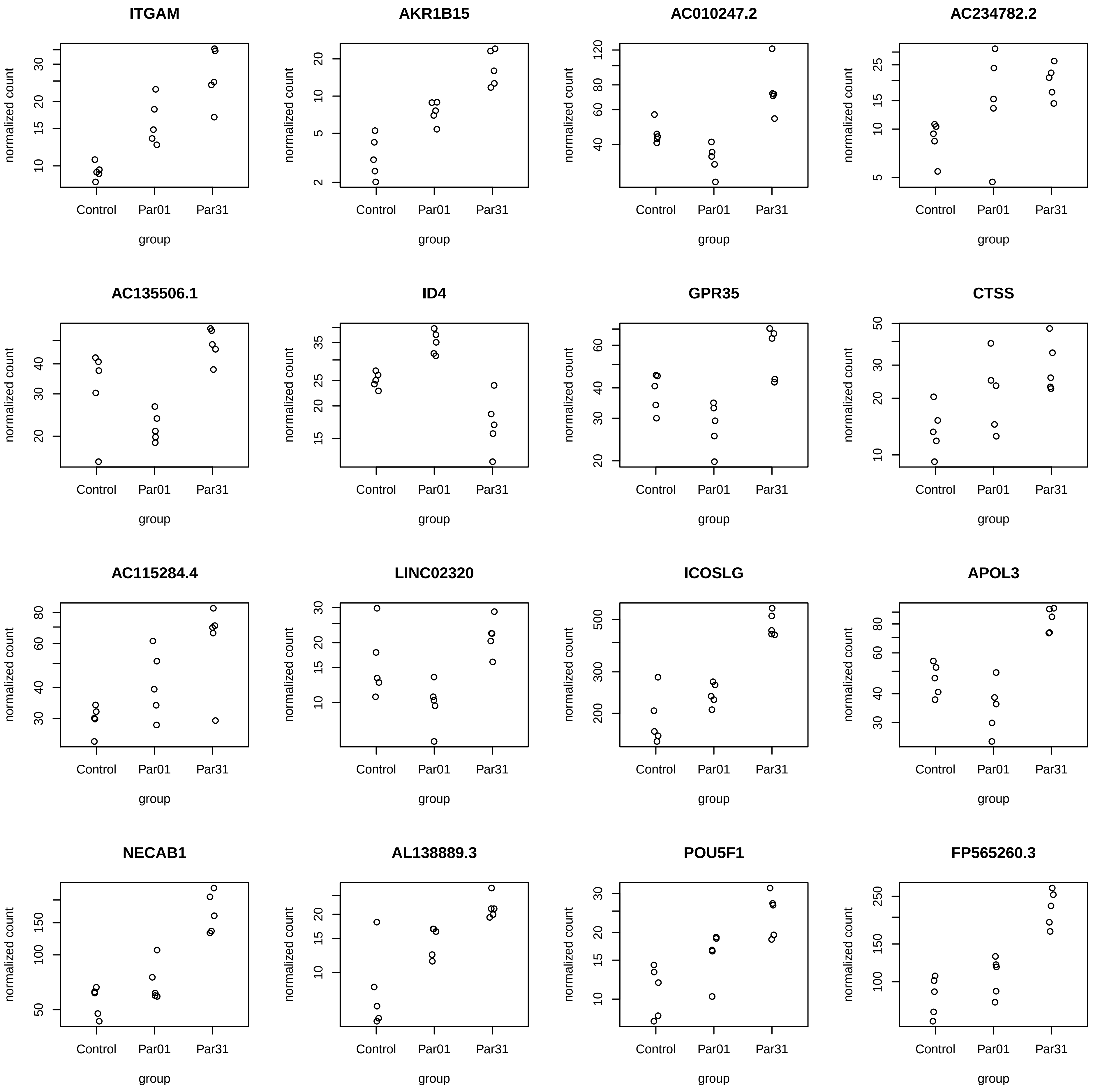

ITGAM
AKR1B15
AC010247.2
AC234782.2
AC135506.1
ID4
GPR35
CTSS
AC115284.4
LINC02320
ICOSLG
APOL3
NECAB1
AL138889.3
POU5F1
FP565260.3
normalized count
group
Control
Par01
Par31

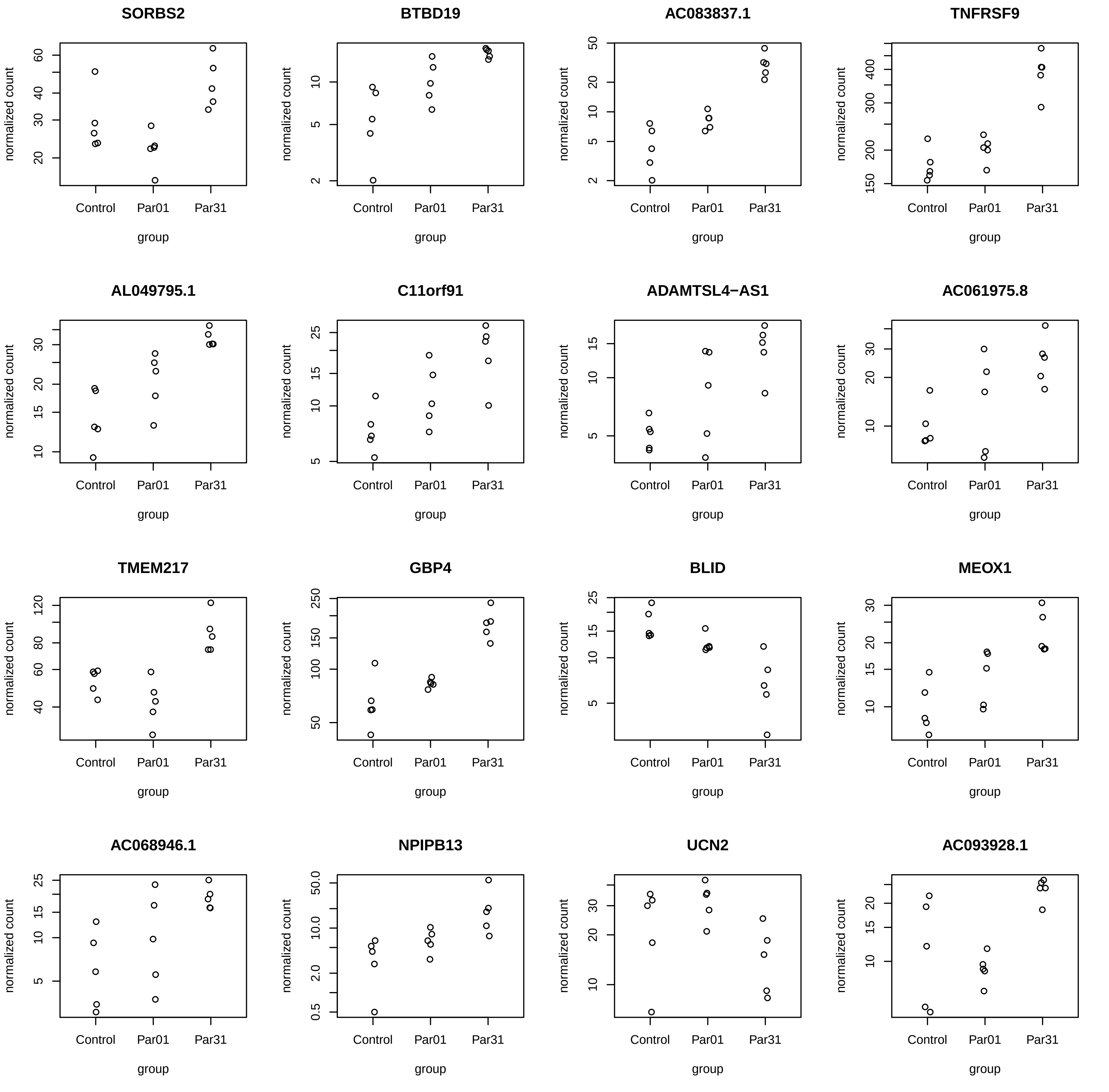
SORBS2
normalized count
20
30
40
60
Control
Par01
Par31
group
BTBD19
2
5
10
AC083837.1
2
5
10
20
50
TNFRSF9
150
200
300
400
AL049795.1
10
15
20
30
C11orf91
5
10
15
25
ADAMTSL4−AS1
5
10
15
AC061975.8
10
20
30
TMEM217
40
60
80
120
GBP4
50
100
150
250
BLID
5
10
15
25
MEOX1
10
15
20
30
AC068946.1
5
10
15
25
NPIPB13
0.5
2.0
10.0
50.0
UCN2
10
20
30
AC093928.1
10
15
20

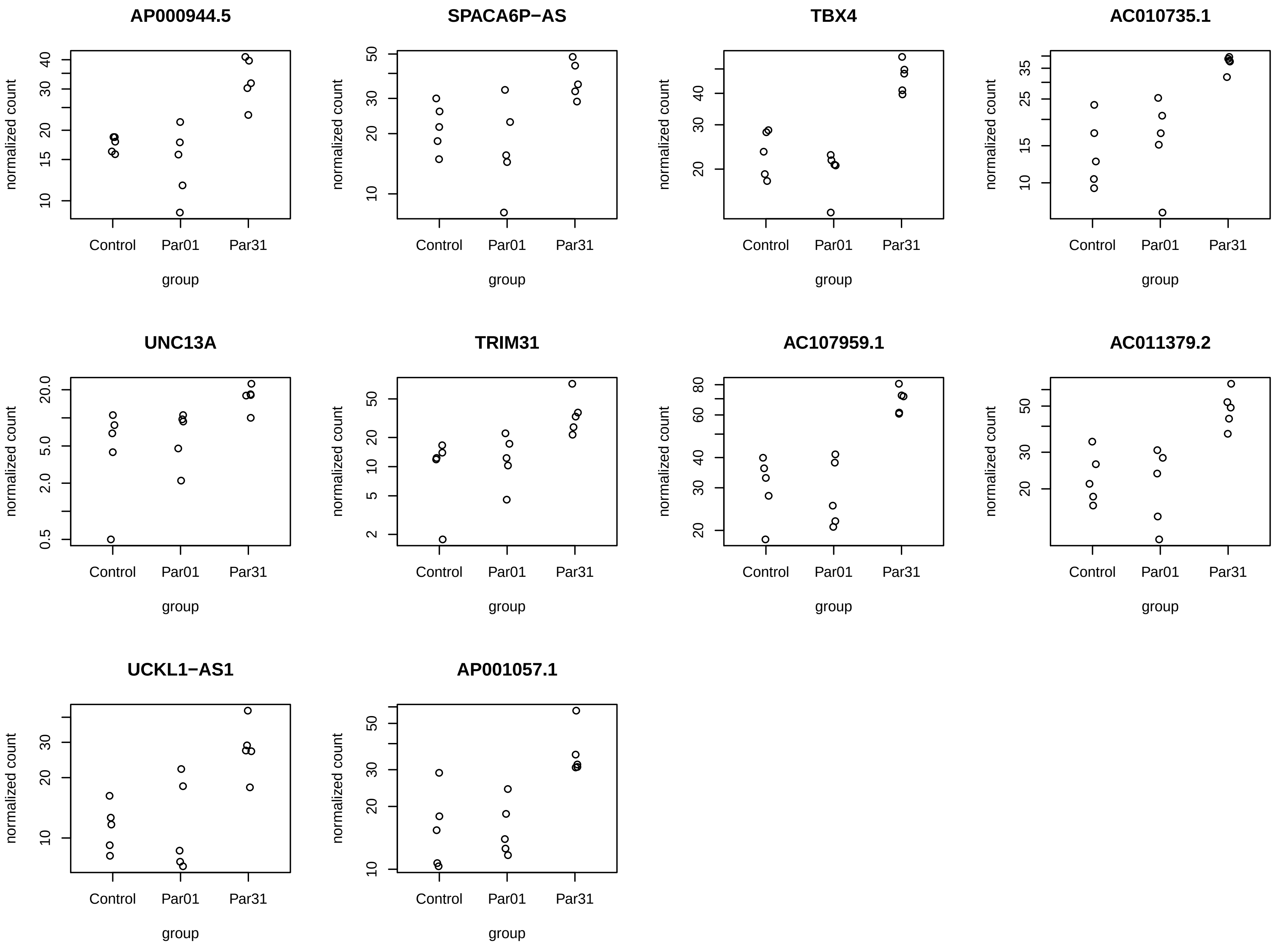

Figure S2: Boxplots of all 298 genes differentially expressed in the PC-3 PF RNAseq experiment.

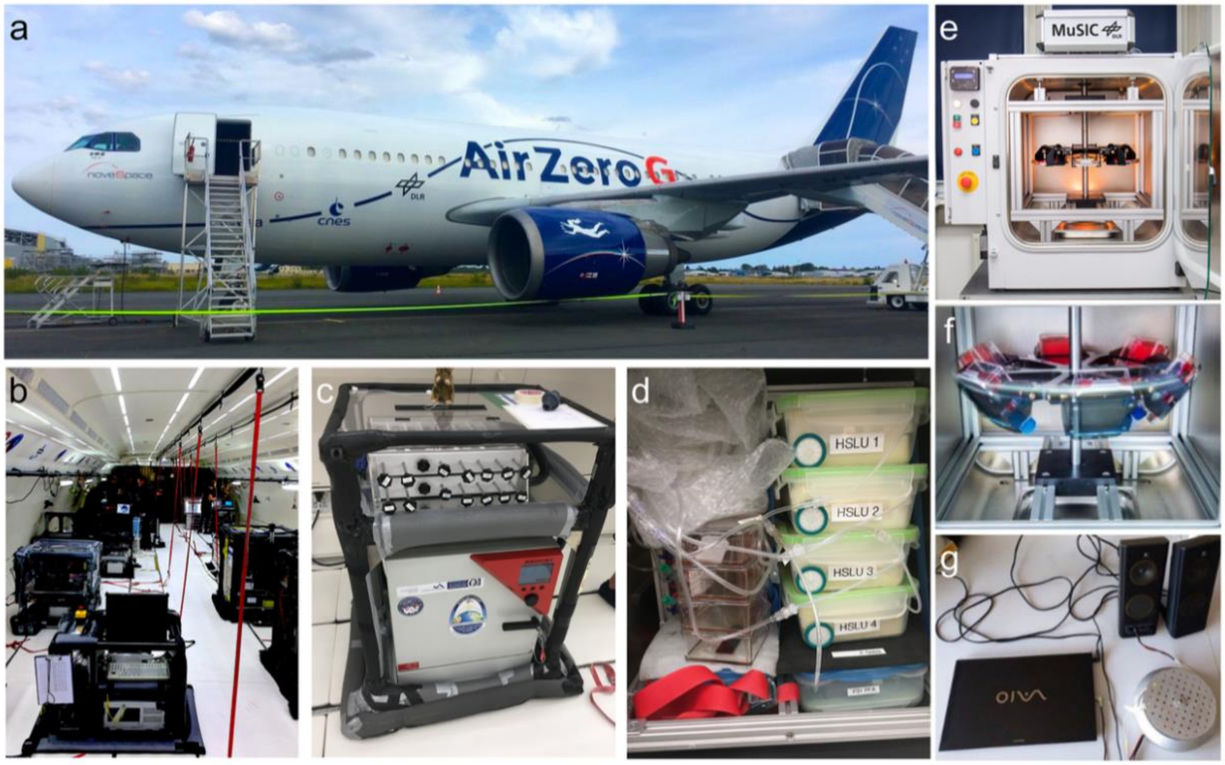

Figure S3. Experiment hardware setup. A: Airbus 310 aircraft used for the 34th DLR parabolic flight campaign. **B:** View in the aircraft, equipped with the experiment racks of several science teams. **C:** The rack with the incubator used for the experiment. **D:** Inside of the incubator prior to take off of the flight. **E:** The Multi Sample Incubator Centrifuge (MuSIC) of DLR (Gravitational Biology) Cologne, placed in an incubator on ground. **F:** Samples within the swing-out device on the MuSIC centrifuge during acceleration. **G**: The Vibraplex device built by DLR (Gravitational Biology), Cologne, Germany.

Table S1 Demultiplexing statistic of the 10 PF and 5 control samples.

Sample	Barcode sequence	PF Clusters	% Perfect barcode	% One mismatch barcode	Yield (Mbases)	% PF Clusters	% >= Q30 bases	Mean Quality Score
1P 1	GCCAAT	54,026,813	98.33	1.67	10,913	100	93.96	35.94
1P 2	CAGATC	65,954,683	98.59	1.41	13,323	100	93.68	35.87
1P 3	ACTTGA	59,237,051	98.95	1.05	11,966	100	93.85	35.92
1P 4	GATCAG	50,967,453	98.98	1.02	10,295	100	94.05	35.95
1P 5	TAGCTT	32,965,164	98.95	1.05	6,659	100	93.54	35.85
31P 1	GGCTAC	61,988,136	99.24	0.76	12,522	100	93.25	35.8
31P 2	CTTGTA	42,205,855	98.4	1.6	8,526	100	93.36	35.82
31P 3	AGTCAA	67,427,596	98.51	1.49	13,620	100	93.49	35.86
31P 4	AGTTCC	64,146,346	98.84	1.16	12,958	100	93.37	35.81
31P 5	ATGTCA	56,824,184	98.38	1.62	11,478	100	93.14	35.79
Control 1	ACAGTG	42,430,335	98.87	1.13	8,571	100	93.92	35.93
Control 2	ATCACG	58,111,319	98.8	1.2	11,738	100	91.96	35.49
Control 3	CGATGT	82,935,298	98.83	1.17	16,753	100	94.14	35.98
Control 4	TTAGGC	42,112,474	99.13	0.87	8,507	100	93.29	35.82
Control 5	TGACCA	67,660,339	98.73	1.27	13,667	100	93.53	35.85

Table S3. Primer selection for the 32 genes and the housekeeper gene 18S in qPCR

Factor	Primer Name	Sequence 5′–3′
18S rRNA	18s-F	GGAGCCTGCGGCTTAATTT
	18s-R	CAACTAAGAACGGCCATGCA
Actin beta (*ACTB*)	ACTB-F	TGCCGACAGGATGCAGAAG
	ACTB-R	GCCGATCCACACGGAGTACT
RAC alpha serine / threonine protein kinase (*AKT1*)	AKT1-F	CTTCTATGGCGCTGAGATTGTG
	AKT1-R	CAGCATGAGGTTCTCCAGCTT
Caspase 3 (*CASP3*)	CASP3-F	CTCCAACATCGACTGTGAGAAGTT
	CASP3-R	GCGCCAGCTCCAGCAA
Caspase 8 (*CASP8*)	CASP8-F	TGCAAAAGCACGGGAGAAAG
	CASP8-R	CTCTTCAAAGGTCGTGGTCAAAG
Caspase 9 (*CASP9*)	CASP9-F	CTCCAACATCGACTGTGAGAAGTT
	CASP9-R	GCGCCAGCTCCAGCAA
Caveolin 2 (*CAV2*)	CAV2-F	GATCCCCACCGGCTCAAC
	CAV2-R	CACCGGCTCTGCGATCA
Cadherin-1 (*CDH1*)	CDH1-F	GCTGGACCGAGAGAGTTTCC
	CDH1-R	CAGCTGTTGCTGTTGTGCTT
Collagen 1 alpha 1 (*COL1A1*)	COL1A1-F	ACGAAGACATCCCACCAATCAC
	COL1A1-R	CGTTGTCGCAGACGCAGAT
Endothelial growth factor (*EGF*)	EGF-F	TGCCAGCTGCACAAATACAGA
	EGF-R	TCTTACGGAATAGTGGTGGTCATC
Endothelial growth factor receptor (*EGFR*)	EGFR-F	TTGCCGCAAAGTGTGTAACG
	EGFR-R	GAGATCGCCACTGATGGAGG
Ezrin (*EZR*)	EZR-F	GCAATCCAGCCAAATACAACTG
	EZR-R	CCACATAGTGGAGGCCAAAGTAC
Vascular endothelial growth factor receptor 2 (*FLK1*)	FLK1-F	TCTTCTGGCTACTTCTTGTCATCATC
	FLK1-R	GATGGACAAGTAGCCTGTCTTCAGT
Vascular endothelial growth factor receptor 1 (*FLT1*)	FLT1-F	CCCTCGCCGGAAGTTGTAT
	FLT1-R	GATAATTAACGAGTAGCCACGAGTCAA
Fibronectin (*FN1*)	FN1-F	AGATCTACCTGTACACCTTGAATGACA
	FN1-R	CATGATACCAGCAAGGAATTGG
Interleukin 6 (*IL6*)	IL6-F	CGGGAACGAAAGAGAAGCTCTA
	IL6-R	GAGCAGCCCCAGGGAGAA
Interleukin 8 (*CXCL8*)	IL8-F	TGGCAGCCTTCCTGATTTCT
	IL8-R	GGGTGGAAAGGTTTGGAGTATG
Cytokeratin 8 (*KRT8*)	KRT8-F	GATCTCTGAGTGAACCGGAACA
	KRT8-R	GCTCGGCATCTGCAATGG
Laminin alpha 3 (*LAMA3*)	LAMA3-F	AAAGCAAGAAGTCAGTCCAGC
	LAMA3-R	TCCCATGAAGACCATCTCGG
Laminin beta 2 (*LAMB2*)	LAMB2-F	TGCTCATGGTCAATGCTAATCTG
	LAMB2-R	TCTATCAATCCTCTTCCTTGGACAA
Matrix metalloproteinase 9 (*MMP9*)	MMP9-F	CCTGGAGACCTGAGAACCAATC
	MMP9-R	TTCGACTCTCCACGCATCTCT
Moesin (*MSN*)	MSN-F	GAAATTTGTCATCAAGCCCATTG
	MSN-R	CCATGCACAAGGCCAAGAT
Mechanistic target of	MTOR-F	ATCTTGGCCATAGCTAGCCTC

rapamycin (*MTOR*)	MTOR-R	ACAACTGGGTCATTGGAGGG
Osteopontin (*OPN*/*SPP1*)	OSP-F	CGAGGTGATAGTGTGGTTTATGGA
	OSP-R	CGTCTGTAGCATCAGGGTACTG
Plasminogen activator inhibitor 1 (*PAI1*)	PAI1-F	AGGCTGACTTCACGAGTCTTTCA
	PAI1-R	CACTCTCGTTCACCTCGATCTTC
Phosphatidylinositol 4,5 bisphosphate 3 kinase catalytic subunit beta (*PIK3CB*)	PIK3CB-F	AGAAAAGTTTGGCCGGTTCC
	PIK3CB-R	GCAGTCAACATCAGCGCAAA
Radexin (*RDX*)	RDX-F	GAAAATGCCGAAACCAATCAA
	RDX-R	GTATTGGGCTGAATGGCAAATT
Transforming growth factor beta (*TGFB1*)	TGFB1-F	CACCCGCGTGCTAATGGT
	TGFB1-R	AGAGCAACACGGGTTCAGGTA
TIMP metallopeptidase inhibitor 1 (TIMP1)	TIMP1-F	GCCATCGCCGCAGATC
	TIMP1-R	GCTATCAGCCACAGCAACAACA
Talin 1 (*TLN1*)	TLN1-F	GATGGCTATTACTCAGTACAGACAACTGA
	TLN1-R	CATAGTAGACTCCTCATCTCCTTCCA
Tubulin beta (*TUBB*)	TUBB-F	CTGGACCGCATCTCTGTGTACTAC
	TUBB-R	GACCTGAGCGAACAGAGTCCAT
Vinculin (*VCL*)	VCL-F	GTCTCGGCTGCTCGTATCTT
	VCL-R	GTCCACCAGCCCTGTCATTT
Vascular Endothelial Growth Factor A (*VEGFA*)	VEGFA	CTACCTCCACCATGCCAAGTG
	VEGFA	GCGCTGATAGACATCCATGAAC
